# SARS-CoV-2 Vaccination Responses in Anti-CD20-Treated Progressive Multiple Sclerosis Patients Show Immunosenescence in Antigen-Specific B and T Cells

**DOI:** 10.3390/vaccines12080924

**Published:** 2024-08-17

**Authors:** Sara De Biasi, Alin Liviu Ciobanu, Elena Santacroce, Domenico Lo Tartaro, Gianluca Degliesposti, Miriam D’Angerio, Maristella Leccese, Martina Cardi, Tommaso Trenti, Michela Cuccorese, Lara Gibellini, Diana Ferraro, Andrea Cossarizza

**Affiliations:** 1 Department of Medical and Surgical Sciences for Children & Adults, University of Modena and Reggio Emilia, 41125 Modena, Italyandrea.cossarizza@unimore.it (A.C.); 2 AOU Policlinico di Modena, Neurology Unit, Department of Biomedical, Metabolic and Neuroscience, University of Modena and Reggio Emilia, 41124 Modena, Italy; 3 AOU Policlinico di Modena, Diagnostic Hematology and Clinical Genomics, Department of Laboratory Medicine and Pathology, 41124 Modena, Italy; 4 National Institute for Cardiovascular Research, 40126 Bologna, Italy

**Keywords:** multiple sclerosis, immune response, vaccines, phenotyping, biomarkers, flow cytometry, immunosenescence

## Abstract

Clinical, pathological, and imaging evidence in multiple sclerosis (MS) shows that inflammation starts early and progresses with age. B cells play a central role in this process, contributing to cytokine production, defective regulatory functions, and abnormal immunoglobulin production, even in the central nervous system. Anti-CD20 (aCD20) therapies, which deplete CD20^+^ B cells, are largely used in the treatment of both relapsing remitting (RR) and progressive (PR) forms of MS. Although effective against MS symptoms and lesions detectable by magnetic resonance imaging, aCD20 therapies can reduce the immune response to COVID-19 vaccination. By using high-parameter flow cytometry, we examined the antigen-specific (Ag^+^) immune response six months post-third COVID-19 mRNA vaccination in MS patients with RR and PR forms on aCD20 therapy. Despite lower Ag^+^ B cell responses and lower levels of anti-SARS-CoV2, both total and neutralizing antibodies, RR and PR patients developed strong Ag^+^ T cell responses. We observed similar percentages and numbers of Ag^+^ CD4^+^ T cells and a high proportion of Ag^+^ CD8^+^ T cells, with slight differences in T cell phenotype and functionality; this, however, suggested the presence of differences in immune responses driven by age and disease severity.

## 1. Introduction

Clinical, pathological, and imaging evidence in patients affected by multiple sclerosis (MS) indicates that inflammatory activity begins early in the disease and progresses gradually with the gender and age of the patient [1,2,3,4,5,6,7,8,9]. This persistent inflammation drives the continuous progression of disability, occurring independently of clinical and radiological relapses. The pathological events responsible for this “chronic” worsening are closely linked to the early accumulation of compartmentalized inflammation within the central nervous system (CNS) [10]. This immune system-driven inflammation is predominantly mediated by B cells, T cells, and glial cells, which perpetuate inflammation and exert toxicity on neighboring neurons. This process is driven by diffuse low-grade inflammation and self-sustaining neuronal deregulation [11]. Over the past two decades, it has been pointed out that, particularly regarding the B cell subset, MS patients exhibit various B cell abnormalities, such as increased pro-inflammatory cytokine production, impaired B cell regulatory functions, and the development of tertiary lymphoid-like structures in the CNS [12], likely causing abnormal immunoglobulin production in the cerebrospinal fluid [13]. Moreover, B cells influence peripheral immune responses by presenting antigens to T cells, thus promoting T-cell activation and proliferation [14].

Given the detrimental role of B cells in MS, anti-CD20 (aCD20) therapies are widely used and are effective and relatively safe. These therapies have revolutionized MS treatment by effectively managing clinical symptoms and lesions identified by magnetic resonance imaging, establishing them as a cornerstone of disease-modifying treatments. Indeed, the clinical efficacy of B cell depletion strategies also prevents new lesion formation [15]. The infusion of aCD20 monoclonal antibodies promotes a depletion of CD20^+^ B cells within hours, primarily occurring in the liver [16]. This depletion typically reaches its nadir around 8 weeks and can be maintained for several weeks to months, depending on the dosage and characteristics of the specific anti-CD20 monoclonal antibody [15].

Currently used aCD20 drugs comprise ocrelizumab, ofatumumab, and rituximab [17]. Ocrelizumab and ofatumumab are both approved for relapsing-remitting MS (RRMS), ocrelizumab has also been approved for primary progressive (PP) MS and Rituximab is widely used as off-label treatment for both RR and progressive forms of MS. From an immunological point of view, RR and PR patients show some differences but also some similarities. The clinical course of MS is highly heterogeneous, leading to various classification attempts based on clinical features like exacerbations, remissions, and progression [18]. Both forms show reduced expression of various adhesion molecules and elevated plasma levels of soluble ICAM-1 and L-selectins. This suggests that the movement of autoreactive leukocytes across the blood-brain barrier is key to the development of MS [19]. Furthermore, progressive MS shows a marked skewed phenotype Th1, T cytotoxic type 1, and Th17 cells in peripheral blood. However, mitochondrial function and metabolic alterations in T cell subpopulations are regulated differently in these forms [20,21].

Recent findings indicate that in ocrelizumab-treated MS patients, B cell depletion does not hinder the development of enduring anti-SARS-CoV-2 T cell responses. These responses are comparable to those in ocrelizumab-treated MS patients without SARS-CoV-2 infection and non-MS individuals with or without the infection. Notably, this response is marked by higher percentages of T memory stem cells, indicating the establishment of long-term memory [22].

However, since the beginning of COVID-19 vaccination campaign, it has been evident that MS patients receiving disease-modifying therapies (DMTs) might be unable to mount a complete immune response against breakthrough COVID-19 infections. This concern was particularly relevant for some DMTs, especially aCD20 agents, which can significantly impact antibody production and reduce the amount of antigen-specific B cells [21,23,24,25,26,27].

A recent study compared anti-SARS-CoV-2 spike antibody titers at 6- and 12-months post-vaccination between two groups: patients vaccinated before initiating aCD20 therapy (i.e., before therapy switch) and those vaccinated while on aCD20 therapy. The findings suggest that patients vaccinated while on aCD20 therapy exhibited weaker immune responses and decreased booster efficacy compared to those vaccinated before starting aCD20 therapy [28].

In this study, we used high-parameter flow cytometry to thoroughly examine the antigen-specific (Ag^+^) immune response six months after the third dose of vaccination against SARS-CoV-2 in two groups of MS patients: those with RR and PR forms, both receiving aCD20 therapy. The results were compared to those obtained in age-matched healthy donors. We found that, although the Ag^+^ B cell response was lower in patients treated with aCD20, both RR and PR patients developed a robust Ag^+^ T cell response. Specifically, we observed similar percentages and absolute numbers of Ag^+^ CD4^+^ T cells in both patient groups, alongside a high proportion of Ag^+^ CD8^+^ T cells. However, in MS patients, the phenotype and functionality of the antigen-specific T cells exhibited slightly different immunological characteristics, reflecting differences in the responses to aCD20 therapy.

## 2. Methods

### 2.1. Patient Selection

The study included 23 patients diagnosed with multiple sclerosis (MS), 10 with the relapsing-remitting (RR) form 13 with progressive form (PR), all undergoing anti-CD20 treatment. Additionally, there were 13 healthy donors (HD) divided into two groups based on the median age: 7 healthy donors aged 35 years (median) to be compared to RR patients (HD_RR) and 6 healthy donors aged 57.5 years (median) to be used for comparison to PR patients (HD_PR). Demographic, clinical, and immunological data of RR patients and HD are taken from [21]. Detailed demographic and clinical characteristics for these individuals, including the type of third-dose vaccine they were administered, and the median range of time since their last DMT administration, are thoroughly documented in Table 1.

To be eligible for inclusion in the study, patients needed to meet specific criteria: (a) a confirmed diagnosis of RR or PR (either PP or secondary progressive) multiple sclerosis and (b) a history of treatment with anti-CD20 therapy, with each patient having undergone at least two infusion cycles of either rituximab or ocrelizumab. In accordance with routine clinical practice, these MS patients received their vaccination against SARS-CoV-2 at a strategically timed interval—at least six weeks prior to a subsequent anti-CD20 infusion or at least three months following their most recent infusion. This timing was critical to avoid potential interference with the immune response to the vaccine.

Exclusion criteria for the study included any recent treatment with steroids within the preceding six weeks, as steroid use could impact immune function, and a history of COVID-19 infection prior to vaccination, to ensure that the vaccine’s effects could be assessed without the confounding influence of previous infection. The study protocol was thoroughly reviewed and approved by the relevant ethical committees to ensure compliance with ethical standards and guidelines. Specifically, the local Ethical Committee (Comitato Etico dell’Area Vasta Emilia Nord) approved the study under protocol number 199/2022 on 24 May 2020. Additionally, the University Hospital Committee (Direzione Sanitaria dell’Azienda Ospedaliero Universitaria di Modena) provided approval under protocol number 5974 on 24 February 2023. All participants, including healthy donors, gave their written informed consent in accordance with the Helsinki Declaration.

### 2.2. Blood Collection and Isolation of Mononuclear Cells

Blood samples, up to 30 mL per patient, were collected using vacuettes containing ethylenediamine-tetraacetic acid (EDTA) as an anticoagulant. The blood samples were processed immediately after collection to ensure the highest cell viability and the accuracy of subsequent analyses. Peripheral blood mononuclear cells (PBMC) were isolated from these samples using the Ficoll-Hypaque density gradient centrifugation method, following standard laboratory procedures [29]. Cryopreserved PBMC were stored in liquid nitrogen in a medium consisting of fetal bovine serum (FBS) supplemented with 10% dimethyl sulfoxide (DMSO). Plasma samples obtained during blood collection were stored at −80 °C until the measure of the levels of antibodies against SARS-CoV-2.

### 2.3. Activation Induced Cell Marker Assay (AIM) and T Cell Phenotype

Isolated PBMCs were first thawed and allowed to rest for a period of six hours to ensure cell recovery before further manipulation. Following this period, a CD40-blocking antibody (final concentration of 0.5 μg/mL) from Miltenyi Biotec (Bergisch Gladbach, Germany) was added to the cell cultures [30]. This antibody was added 15 min prior to Ag stimulation to inhibit any unwanted activation through the CD40 pathway, which could otherwise affect the accuracy of the assay results.

PBMCs were stimulated using 15 mer peptides with 11 amino acid overlaps, covering the entire sequence of the Wuhan strain SARS-CoV-2 Spike glycoprotein (PepTivator SARS-CoV-2 Prot_S complete, Miltenyi Biotec) at a final concentration of 1 μg/mL. This peptide pool was used with the presence of 1 μg/mL of anti-CD28/49d antibody (Miltenyi Biotec) to give co-stimulatory signals necessary for T cell activation. PBMCs were incubated for 18 h at 37 °C in a humidified atmosphere containing 5% CO_2_. The complete culture medium used for this assay consisted of RPMI 1640, supplemented with 10% fetal bovine serum (FBS), 1% L-glutamine, sodium pyruvate, nonessential amino acids, antibiotics, 0.1 M HEPES, and 55 μM β-mercaptoethanol, and 0.02 mg/mL DNAse. For each experimental condition, a corresponding unstimulated sample was prepared to serve as a negative control. This is critical for accurately identifying and gating activated T cells by comparing stimulated samples to their baseline, unstimulated states. After the stimulation period, cells were washed with phosphate-buffered saline (PBS) and then stained with PromoFluor IR-840 (PromoCell, Heidelberg, Germany) for 20 min at room temperature (RT). This initial staining step helps to exclude dead cells from the analysis, ensuring that only live cells are assessed in subsequent steps. Following the initial staining, cells were washed with FACS buffer (PBS supplemented with 2% FBS) and stained with fluorochrome-labelled monoclonal antibodies (mAbs) targeting these surface molecules: CXCR5-BUV661, CCR6-BUV496, and CXCR3-BV785. This staining was performed for 30 min at 37 °C, in an atmosphere containing 5% CO_2_. Subsequently, cells were washed again with FACS buffer and stained for an additional 20 min at RT with the Duraclone IM T cell panel (Beckman Coulter, Brea, CA, USA). This panel includes a comprehensive set of markers: CD45-Krome Orange, CD3-APC-A750, CD4-APC, CD8-AF700, CD27-PC7, CD57-Pacific Blue, CD279 (PD1)-PC5.5, CD28-ECD, CCR7-PE, and CD45RA-FITC. Additionally, to further characterize activation-induced cell markers, three others fluorescent mAbs were incorporated: CD69-BV650, CD137-BUV395, and CD95-BV605 [31].

All samples were acquired using a CytoFLEX LX flow cytometer (Beckman Coulter). A minimum of 1,000,000 cells per sample were collected to ensure robust statistical analysis and reliable detection of rare cell populations. The data acquisition process involved the use of optimized gating strategies to accurately identify CD4^+^ and CD8^+^ T cell populations. These gating strategies are represented in detail in the Supplementary Figures S1 and S2. The reagents and antibodies used for T cell phenotyping are listed in Supplementary Table S1. All monoclonal antibodies added to the Duraclone IM T cell panel were titrated on human PBMCs beforehand to determine the optimal concentrations, ensuring the best signal-to-noise ratio and accurate identification of T cell subsets.

### 2.4. Detection of SARS-CoV-2-Specific B Cells

Thawed PBMCs were initially washed twice with RPMI 1640 medium supplemented with 10% FBS, 1% L-glutamine, sodium pyruvate, nonessential amino acids, antibiotics, 0.1 M HEPES, 55 μM β-mercaptoethanol, and 0.02 mg/mL DNase. Following these washes, PBMCs were stained with the viability marker PromoFluor IR-840 (Promokine, by PromoCell, Heidelberg, Germany) for 20 min at room temperature (RT) in PBS. Next, cells were washed with PBS to remove any unbound dye and stained with streptavidin-AF700 (ThermoFisher Scientific, Eugene, OR, USA) for 15 min at RT. This step was implemented to eliminate false positives by binding to any non-specific biotinylated antibodies, ensuring that only specific SARS-CoV-2 B cells were detected. Following a wash with FACS buffer (PBS supplemented with 2% FBS), the cells were stained with biotinylated full-length SARS-CoV-2 spike protein (R&D Systems, Minneapolis, MN, USA), labelled with different streptavidin-fluorophore conjugates. Specifically, the full-length biotinylated spike protein was mixed and incubated with streptavidin-BUV661 (Becton Dickinson, San José, CA, USA) or streptavidin-BV650 (BioLegend, San Diego, CA, USA) at a 6:1 mass ratio for 15 min at RT [32].

All samples were stained with these biotinylated streptavidin conjugates for one hour at 4 °C to allow for optimal binding. After staining, the cells were washed with FACS buffer to remove excess reagents and reduce background fluorescence. The final staining step involved the use of the DuraClone IM B cells panel (Beckman Coulter, Brea, CA, USA) for 20 min at RT. This panel included the following lyophilized, directly conjugated monoclonal antibodies (mAbs): anti-IgD-FITC, CD21-PE, CD19-ECD, CD27-PC7, CD24-APC, CD38-AF750, anti-IgM-PB, and CD45-KrO. In addition, we added the following drop-in antibodies: CD71-BUV395, CD20-BV785, anti-IgG-BUV496, and anti-IgA-PerCP-Vio700. The prepared samples were then acquired using a CytoFLEX LX flow cytometer (Beckman Coulter). A minimum of 1,000,000 cells per sample were collected to ensure robust statistical analysis and reliable detection of rare cell populations. All reagents and antibodies used for B cell phenotyping are listed in Supplementary Table S2. All monoclonal antibodies added to the DuraClone IM B cells panel were titrated on human PBMCs prior to use. This titration process was essential to determine the optimal antibody concentrations, ensuring the best signal-to-noise ratio and accurate identification of antigen-positive (Ag^+^) and antigen-negative (Ag^−^) B cells. The detailed gating strategy used to distinguish these populations is provided in Supplementary Figure S3.

### 2.5. Intracellular Cytokine Staining (ICS)

Isolated PBMCs were thawed and allowed to rest for six hours to ensure cell viability. Following this rest period, PBMCs were stimulated with a pool of lyophilized peptides that encompass the entire protein coding sequence (amino acids 5-1273) of the SARS-CoV-2 spike glycoprotein (“S”) (PepTivator SARS-CoV-2 Prot_S Complete, Miltenyi Biotec, Teterow, Germany) at a final concentration of 1 μg/mL. Additionally, 1 μg/mL of anti-CD28/49d (Becton Dickinson) was added to provide necessary co-stimulatory signals. The PBMCs were incubated with these peptides for 16 h at 37 °C in a 5% CO_2_ atmosphere, using a complete culture medium composed of RPMI 1640 supplemented with 10% FBS, 1% each of L-glutamine, sodium pyruvate, non-essential amino acids, antibiotics, 0.1 M HEPES, 55 mM β-mercaptoethanol, and 0.02 mg/mL DNAse. For each experimental condition, an unstimulated control sample was prepared to serve as a negative control. This ensures that any detected cytokine production can be attributed to the specific stimulation. During the incubation, protein transport inhibitors brefeldin A (Golgi Plug, Becton Dickinson) and monensin (Golgi Stop, Becton Dickinson) were added to facilitate intracellular cytokine accumulation, along with a titrated concentration of CD107a-PE (BioLegend, San Diego, CA, USA). After stimulation, the cells were washed with PBS and stained with LIVE/DEAD Fixable Aqua (ThermoFisher Scientific, USA) for 20 min at room temperature to differentiate live cells from dead cells. The cells were then washed with FACS buffer (PBS supplemented with 2% FBS) and stained with the following surface monoclonal antibodies (mAbs) CD3-PE.Cy5, CD4-AF700, and CD8-APC.Cy7 (BioLegend). After surface staining, the cells were washed again with FACS buffer and then fixed and permeabilized using the Cytofix/Cytoperm buffer set (Becton Dickinson) to allow for intracellular cytokine detection. The permeabilized cells were subsequently stained with the following pre-titrated mAbs: IL-17-PE-Cy7, TNF-BV605, IFN-γ-FITC, IL-2-APC, and GRZB-BV421 (all from BioLegend). The samples were then analyzed using an Attune NxT acoustic cytometer (ThermoFisher Scientific). Detailed information regarding the mAb titers, clones, catalogue numbers, and the types of fluorochromes used in the panel can be found in Supplementary Table S3. The gating strategy used to identify and analyze intracellular cytokine production in CD4^+^ and CD8^+^ T lymphocytes is represented in detail in Supplementary Figure S4.

### 2.6. Computational Analysis of Flow Cytometry Data

#### 2.6.1. T Cell Analysis

Flow cytometry data files, in the form of Compensated Flow Cytometry Standard (FCS) 3.0, were imported into FlowJo software version 10.7.1 for the initial analysis phase. We employed standard gating strategies to effectively remove doublets, aggregates, and dead cells, ensuring that only single, viable cells were included in subsequent analyses [33]. This step is essential for maintaining the integrity and reliability of the data.

For the ex vivo immunophenotyping of both non-antigen-specific (Ag^−^) and antigen-specific (Ag^+^) T cells within the CD4^+^ and CD8^+^ populations, we focused solely on data derived from stimulated samples. From each sample, we extracted data corresponding to all viable CD4^+^ or CD8^+^ T cells and imported these datasets into R using the flowCore package (version 2.4.0). This process resulted in the analysis of a total of 143,403 cells for CD4^+^ SARS-CoV-2 specificity, and 74,899 cells identified as CD8^+^ SARS-CoV-2-specific T cells.

Further analysis was conducted using the CATALYST package (version 1.17.3) in R. All flow cytometry data underwent a hyperbolic arcsine transformation “arcsinh (x/cofactor)”, where x represents the fluorescence intensity value. This transformation, using manually defined cofactors, normalized the data, and facilitated accurate comparisons across different fluorescence channels.

Clustering and dimensionality reduction were performed using the FlowSOM algorithm (version 2.4.0) and the UMAP algorithm (version 0.2.8.0), respectively. These advanced computational techniques enabled us to identify and visualize distinct T cell subpopulations based on their expression profiles. Specifically, we analysed clusters of Ag^+^ CD4^+^ and CD8^+^ T cells using a comprehensive panel of markers, including CD45RA, CCR7, CD27, CD28, PD-1, CCR6, CXCR3, CXCR5, and CD95. These markers provided detailed insights into the activation and differentiation states of the T cells.

Quality control (QC) procedures for clustering analysis were meticulously carried out and documented to ensure the robustness and reliability of our methodology. The QC results for CD4^+^ and CD8^+^ T cell clustering are presented in Supplementary Figures S7 and S10, respectively.

#### 2.6.2. B Cell Analysis

For B cell analysis, compensated Flow Cytometry Standard (FCS) 3.0 files were similarly imported into FlowJo software version 10.7.1 for initial examination. Standard gating techniques were applied to remove doublets, aggregates, and dead cells, ensuring that only single, viable cells were considered. CD19^+^ B cells were then identified and selected for further analysis. To ensure the specificity of the SARS-CoV-2 analysis, decoy-positive B cells were excluded from the total CD19^+^ B cell population, effectively removing false positives.

For each sample, SARS-CoV-2-specific B cells were identified based on their positive staining for both Spike_streptavidin-BUV661 and Spike_streptavidin-BV650, referred to as Ag^+^ B cells. Cells that did not stain positive for these markers were categorized as non-SARS-CoV-2-specific B cells, referred to as Ag^−^ B cells. This dual staining approach ensured accurate identification of SARS-CoV-2-specific B cells.

Data for Ag^+^ B cells from each sample were then exported and imported into R using the flowCore package (version 2.4.0) for comprehensive computational analysis. Unsupervised analysis was performed on a total of 3042 Ag^+^ B cells using the CATALYST package (version 1.17.3) in R. All flow cytometry data were transformed using a hyperbolic arcsine function “arcsinh (x/cofactor)”, with manually defined cofactors to normalize fluorescence intensity values and facilitate accurate data comparisons.

### 2.7. Statistical Analysis

For the comparison of quantitative variables, we employed the Kruskal–Wallis non-parametric test. To adjust for multiple comparisons and control the False Discovery Rate (FDR), we used the Benjamini and Hochberg correction method. The q-values that achieved statistical significance are indicated in the results. The analysis of cytokine production data was conducted using GraphPad Prism version 8 (GraphPad Software Inc., La Jolla, CA, USA). Background-subtracted data were used to calculate the total percentages of antigen-specific (Ag^+^ CD4^+^ and Ag^+^ CD8^+^) T cells. To examine the polyfunctionality of T cells, the Simplified Presentation of Incredibly Complex Evaluations (SPICE) software, version 6 (kindly provided by Dr. Mario Roederer, Vaccine Research Center, NIAID, NIH, Bethesda, MD, USA) was used. The data for cytokine production are displayed as individual values, along with means and standard errors of the mean (SEM). For polyfunctionality analyses, pie charts depict median values and permutation tests were used for statistical analysis; graphical data are represented as individual values, means, and SEM.

### 2.8. Principal Component Analysis

Principal component analysis (PCA) was performed and visualized using R, leveraging the prcomp function from the stats package (version 3.6.2) and the pca3d package (version 0.1). The analysis included data on proportions, absolute numbers, and clinical parameters, as documented in the Source Data File. Missing data in the dataset were imputed using the missMDA package (version 1.18). The overall contribution of each variable to the first and second principal components (PC1 and PC2) was calculated with the formula [(C1 × Eig1) + (C2 × Eig2)]/(Eig1 + Eig2), where C1 and C2 represent the contributions to PC1 and PC2, respectively, and Eig1 and Eig2 denote the eigenvalues of PC1 and PC2. To determine the Euclidean distance between multiple sclerosis (MS) treatment groups and healthy donors (HD) in the PCA space, we utilized the phenoptr package (version 0.3.2).

## 3. Results

### 3.1. MS Patients Undergoing aCD20 Therapy Develop a CD4^+^ Ag^+^ Specific T Cell Response Similar to Healthy Donors

**Figure 1:**
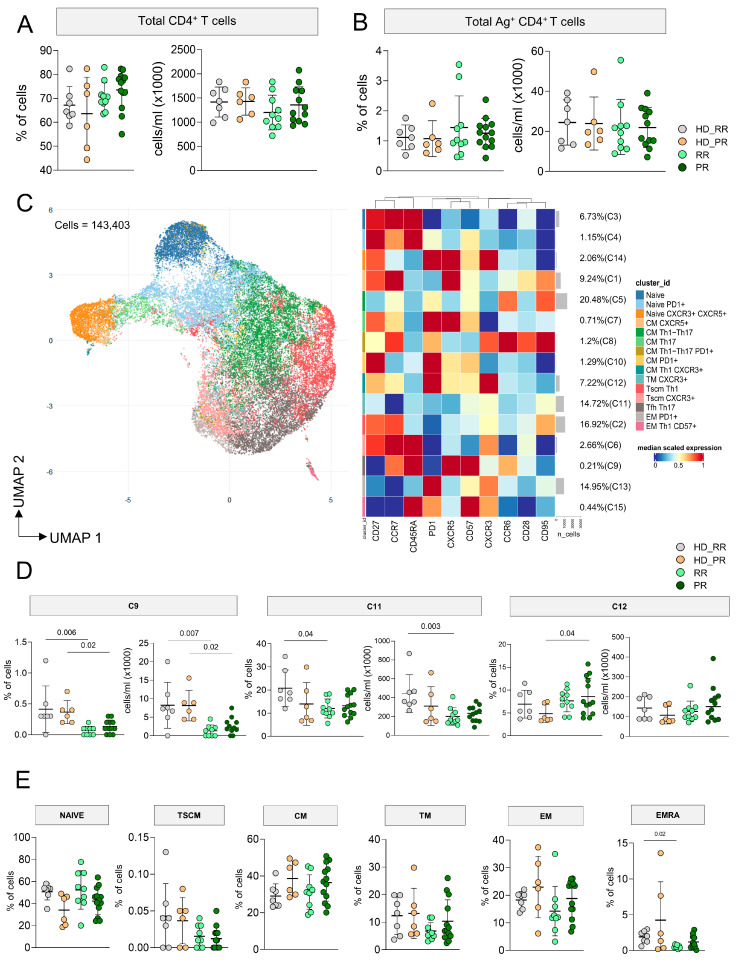
Landscape of antigen-specific CD4^+^ T lymphocytes. (**A**) The panels with the dots report the percentage (**left**) or absolute number (**right**) of CD4^+^ T cells. Bars indicate mean ± SD; one-sided Kruskal–Wallis test with Benjamini–Hochberg correction for multiple comparisons was used for statistical analysis, that revealed no difference among groups. (**B**) Percentage and absolute number of CD4^+^ T cells that recognize SARS-CoV-2 peptides; statistical analysis as in the previous panel. (**C**) Left: phenotype of Ag^+^ CD4^+^ T cells, as revealed by the Uniform Manifold Approximation and Projection (UMAP) of 143,403 cells from healthy donors (HD) and MS patients treated with aCD20. Right: FlowSOM algorithm after the manual metacluster merging evidence 15 cell populations that derive from 10 lineage markers, and that are shown by the heatmap related to the median marker intensities. The colors of the column related to cluster_id are the same of those used to label UMAP clusters. The color in the heatmap is referred to the median of the arcsinhmarker expression (0–1 scaled) calculated over cells from all the samples, where red represents high expression of the marker of interest and blue represents low expression. Light gray bar along the rows (clusters) and values in brackets indicate the relative sizes of the clusters. Cell legend: N naïve, TSCM T stem cell memory, CM central memory, TM transitional memory, EM effector memory, EMRA effector memory re-expressing CD45RA, Tfh T follicular helper cells. (**D**) Proportion of cells present in different clusters. Statistical analysis was performed by using Kruskal–Wallis test with Benjamini–Hochberg correction for multiple comparison. The adjusted *q*-values are reported in the figure. (**E**) Manual gating-obtained proportions of different populations present among CD4^+^ T cells, analyzed as in panel A. (**A**–**E**) Data are related to healthy donors matched with relapsing remitting (RR) patients (HD_RR): *n* = 7; healthy donors matched with progressive (PR) patients HD_PR: *n* = 6; relapsing remitting patients (RR): *n* = 10; progressive patients (PR): *n* = 12.

MS patients undergoing aCD20 therapy, either with RR or PR forms, displayed similar percentages and absolute numbers of CD4^+^ T cells when compared to age-matched HD (Figure 1A). After 18 h of in vitro stimulation with SARS-CoV-2 peptides, taking into account T cells that upregulated CD137 and CD69 (activation induced marker, AIM assay), we were able to investigate the percentages and absolute numbers of Ag^+^ T cells. As for CD4^+^ T cells, we observed similar percentages and absolute numbers of CD4^+^ Ag^+^ T cells as those observed in age-matched HD (Figure 1B).

As far as the phenotype of Ag^+^ T cells was concerned, in particular, their differentiation in terms of Th1, Th0/2, Th17, and circulating follicular helper (Tfh) cells, 143,403 CD4^+^ Ag^+^ T cells, obtained from MS patients and HD, were used for the unsupervised analysis. FlowSOM cell clustering revealed 15 clusters spanning from less differentiated cells (naïve) to terminally differentiated effector memory cells (Figure 1C, Supplementary Figures S5 and S6). Markers such as CD27, CCR7, CD28, CD45RA, CD95 and CD57 were considered for detecting diverse differentiation status such as naïve (CCR7^+^CD45RA^+^CD28^+^CD27^+^CD95^−^), central memory (CCR7^+^CD45RA^−^CD28^+^CD27^+^CD95^+^), effector (CCR7^−^CD45RA^−^CD28^−^CD27^+/−^CD95^+^), transitional memory (CCR7^−^CD45RA^−^CD28^+^CD27^+/−^CD95+), and terminally differentiated cells (CCR7^−^CD45RA^+^CD28^−^CD27^+/−^CD95^+^), the latter with features of senescence (i.e., expressing CD57). Markers like CCR6, CXCR3, and CXCR5 were used to identify cell skewing in terms of Th1 (CXCR3^+^), Th17 (CCR6^+^), Th0/Th2 (CXCR3^−^CCR6^−^), or Tfh (CXCR5^+^PD-1^+^).

Four different main clusters identifying different subpopulations of Ag^+^ T cells account for the 67% of the total Ag^+^ T cells: these clusters were C5 (20.48%) identified as central memory Th17 (CM_th17_), C11 (14.72%) identified as transitional memory (TM_Th0/2_), C13 (14.95%) representing EM expressing PD-1, and C2 (16.92%) representing central memory CM_Th0/2_. C3 and C4 represented two clusters of naïve cells, C14 identified naïve cells expressing CXCR5, C11 were TM_th1_, C9 spotted Tfh, C8 was a cluster of CM_Th1/Th17_ expressing PD-1, C7 is a cluster of CM_Th17_ T cells that express CXCR5^+^, C10 are effector memory (EM) lymphocytes expressing PD-1 and CD27, C12 a cluster of cells CM_Th1_ expressing PD-1, C15 a small cluster of EM_Th1_ expressing CD57. Finally, C14 identified Tfh expressing PD-1 and CXCR3, whose presence positively correlate with neutralizing antibody responses [34].

In general, MS patients displayed slightly lower percentages and absolute numbers of C9, but only RR showed lower absolute numbers, but not percentages, of this cluster when compared to age-matched HD. Similar results were also observed for C13, so lower absolute numbers of EM_th1_ Ag^+^ T cells in MS patients, but more specifically in RR patients when compared to age-matched HD. The percentage of C12 was higher in PR patients if compared to aged-matched HD (Figure 1D). No differences were found as far as all other clusters were concerned (Supplementary Figure S7).

To evaluate if any differences in the differentiation status of Ag^+^ T cells could depend on the differences affecting the non-Ag^+^ CD4^+^ T cell population, by manual gating we investigated the differentiation status of CD4^+^ T cells. We observed similar percentages of naïve, T_SCM_ (CCR7^+^CD45RA^+^ CD28^+^CD27^+^CD95^+^), CM (CCR7^+^CD45RA^−^), TM (CCR7^−^CD45RA^−^CD28^+^), and EM (CCR7^−^CD45RA^−^) cells as far as age-matched donors, and MS patients (HD_RR vs. RR and HD_PR vs. PR) were compared. However, RR patients displayed lower percentages of EMRA (CCR7^−^CD45RA^+^) T cells when compared to age-matched donors (Figure 1E).

### 3.2. Progressive MS Patients Display High Percentage of CD4^+^CD107a^+^ Ag^+^ Specific T Cell

**Figure 2:**
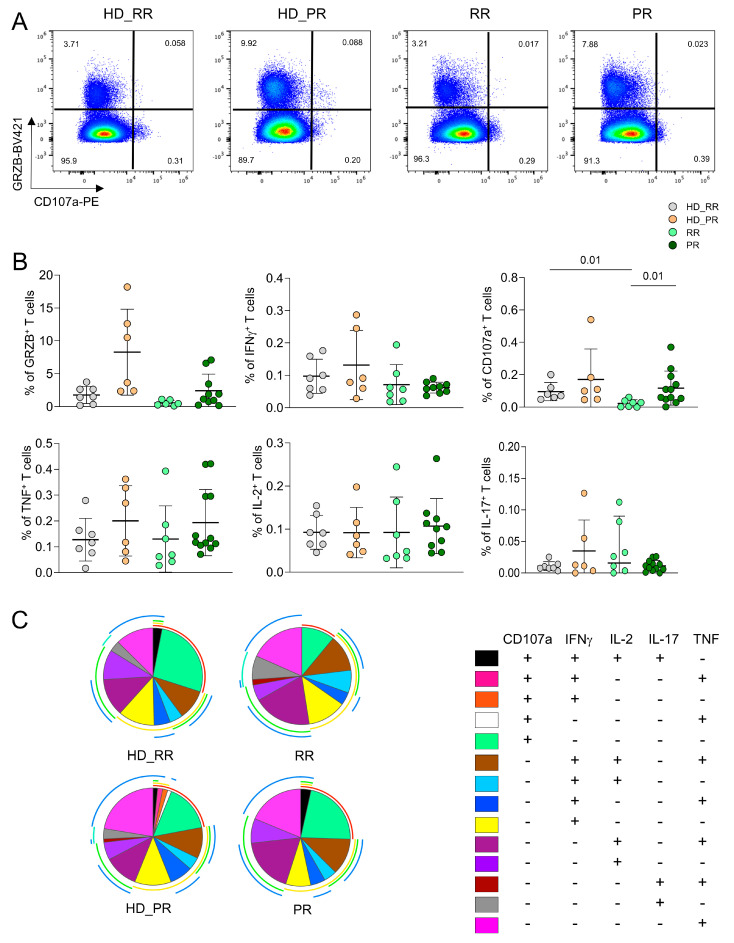
CD4^+^ Ag^+^ T polyfunctional profiles. (**A**) Dot plots obtained by the flow cytometry software Flow-Jo indicate the percentages of CD4^+^ Ag^+^ cells able to produce granzyme B (GRZB) or expressing CD107a. (**B**) Percentage of Ag^+^ CD4^+^ T cells that produce different molecules after in vitro stimulation with SARS-CoV-2 peptides. Analysis was performed as in the Legend to Figure 1. (**C**) Profile of polyfunctional antigen-specific CD4^+^ T cells. Pie charts represent the proportion of Ag^+^ CD4^+^ T cells that contain combinations of CD107a, IL-2, IL-17, IFN-*γ*, and TNF. Each color refers to specific CD4^+^ T cell population as reported in the “polyfunctionality legend” in the bottom right part.

The percentage of CD4^+^ Ag^+^ T cell able to produce different cytokines after in vitro stimulation was assessed by intracellular cytokine staining (ICS) measuring cells producing interferon-g (IFN-g), tumor necrosis factor (TNF), interleukin-(IL)-17, IL-2, and cytotoxic features such as granzyme B (GRZB) and CD107a. Higher percentage of cells expressing CD107a, i.e., those able to degranulate cytotoxic enzymes, was higher in PR patients when compared to RR patients. Moreover, RR patients displayed lower percentage of this population when compared to age-matched HD. Similar percentages of cells producing different features was found among SM and HD groups (Figure 2A). No differences were found also in terms of polyfunctionality, the capability of cells to produce different cytokines at the same time; indeed, both SM patients and HD displayed polyfunctional features (Figure 2B).

### 3.3. Higher Percentage of CD8^+^ Ag^+^ T Cells Characterize Progressive Patients

**Figure 3:**
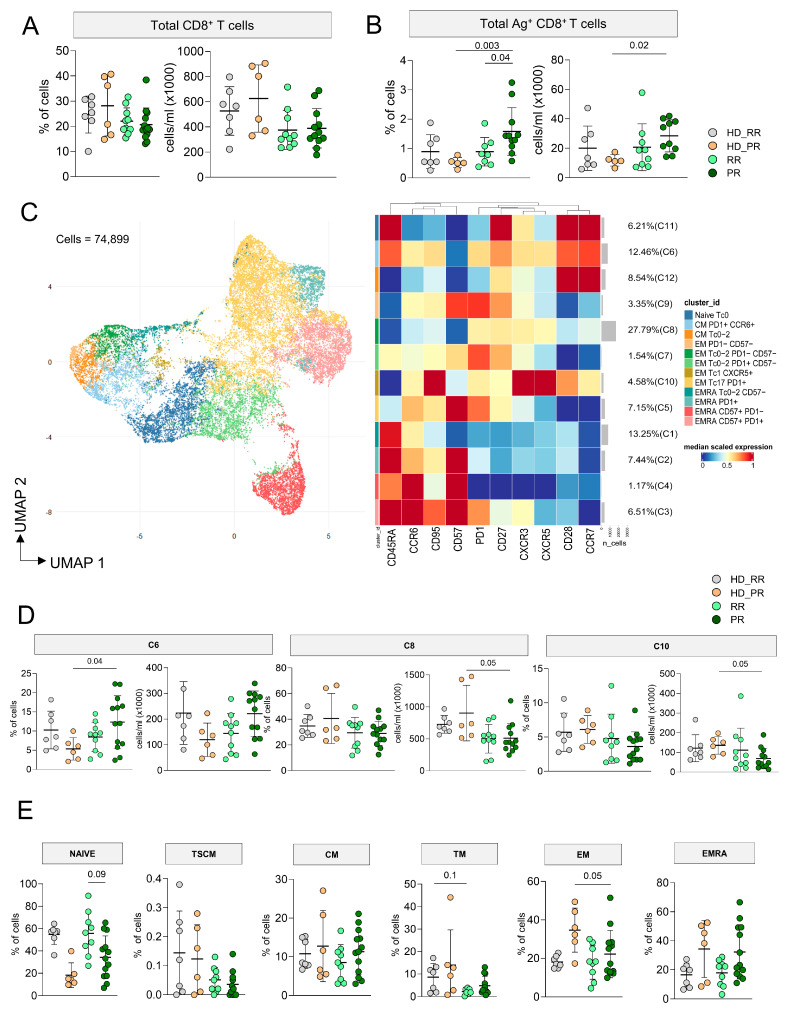
Phenotype of antigen-specific CD8^+^ T lymphocytes. (**A**) The panels with the dots report the percentage (left) or absolute number (right) of CD8^+^ T cells. Bars indicate mean ± SD; one-sided Kruskal–Wallis test with Benjamini–Hochberg correction for multiple comparisons was used for statistical analysis, that revealed no difference among groups. (**B**) Percentage and absolute number of CD8^+^ T cells that recognize SARS-CoV-2 peptides; statistical analysis as in the previous panel. (**C**) Left: phenotype of Ag^+^ CD8^+^ T cells, as revealed by the Uniform Manifold Approximation and Projection (UMAP) of 74,899 cells from healthy donors (HD) and MS patients treated with aCD20. Right: FlowSOM algorithm after the manual metacluster merging evidence 11 cell populations that derive from 10 lineage markers, and that are shown by the heatmap related to the median marker intensities. The colors of the column related to cluster_id are the same of those used to label UMAP clusters. See Figure 1 for further details. Cell legend: N naive, TSCM T stem cell memory, CM central memory, TM transitional memory, EM effector memory, EMRA effector memory re-expressing CD45RA, Tfh T follicular helper cells. (**D**) Proportion of cells present in different clusters. Statistical analysis was performed by using Kruskal–Wallis test with Benjamini–Hochberg correction for multiple comparison. The adjusted q-values are reported in the figure. (**E**) Manual gating-obtained proportions of different populations present among CD4^+^ T cells, analyzed as in panel A. (**A**–**E**) donors and patients as in the Legend to Figure 1.

The percentage and the absolute number of CD8^+^ T cells was similar in MS and HD (Figure 3A), but PR MS patients displayed higher percentage as well as absolute number of CD8^+^ Ag^+^ T cells when compared to age-matched HD (Figure 3B). An amount of 74,899 CD8^+^ Ag^+^ T cells from MS patients and HD were considered to investigate by FlowSOM the phenotype of cells and we identified 12 clusters (Figure 3C, Supplementary Figures S8–S10). A percentage of 27.79% of cells were grouped into C8, a cluster that identified EM_Tc0/2_, PD-1^+^ Ag^+^ T cells. C6, which accounted for the 12.46% of cells, represented CM_Tc17_, PD-1^+^ cells; this cluster was similar to C7. C1 (13.25%) spotted EMRA_Tc0–2_ cells, C2, C3, C4, and C5 identified EMRA_Tc17_ cells, but C3 grouped those EMRA_Tc17_ expressing CD57, PD-1, and CD27. C9 was a cluster of EMTc0/2 expressing CD27, C10 identified CM_Tc1–17_ cells expressing CXCR5. C11 represented naïve cells, while C12 were CM_Tc0–2_.

PR patients displayed higher percentage of C6 (naïve, PD-1^+^ cells), but lower absolute number of C8 (EM_Tc0/2_, PD-1^+^), C10 (EM_Tc17_ CXCR5^+^), and C12 (CM_Tc0–2_) if compared to age-matched HD (Figure 3D). Similar percentages of all other clusters were found in all other group (Supplementary Figure S11).

As for CD4^+^ T cells, to evaluate if any differences in the differentiation status of Ag^+^ T cells could depend on the differences affecting the Ag^−^ CD8^+^ T cell population, by manual gating, we investigated the differentiation status of CD8^+^ T cells. PR MS patients displayed lower percentages of naïve and higher percentages of TM CD8^+^ T cells when compared to RR MS patients. Furthermore, PR MS patients display lower percentage of EM CD8^+^ T cells compared to their HD counterpart (Figure 3E).

### 3.4. CD8^+^ Ag^+^ T Cells from PR Patients Are Much More Polyfunctional If Compared to Age-Matched HD

**Figure 4:**
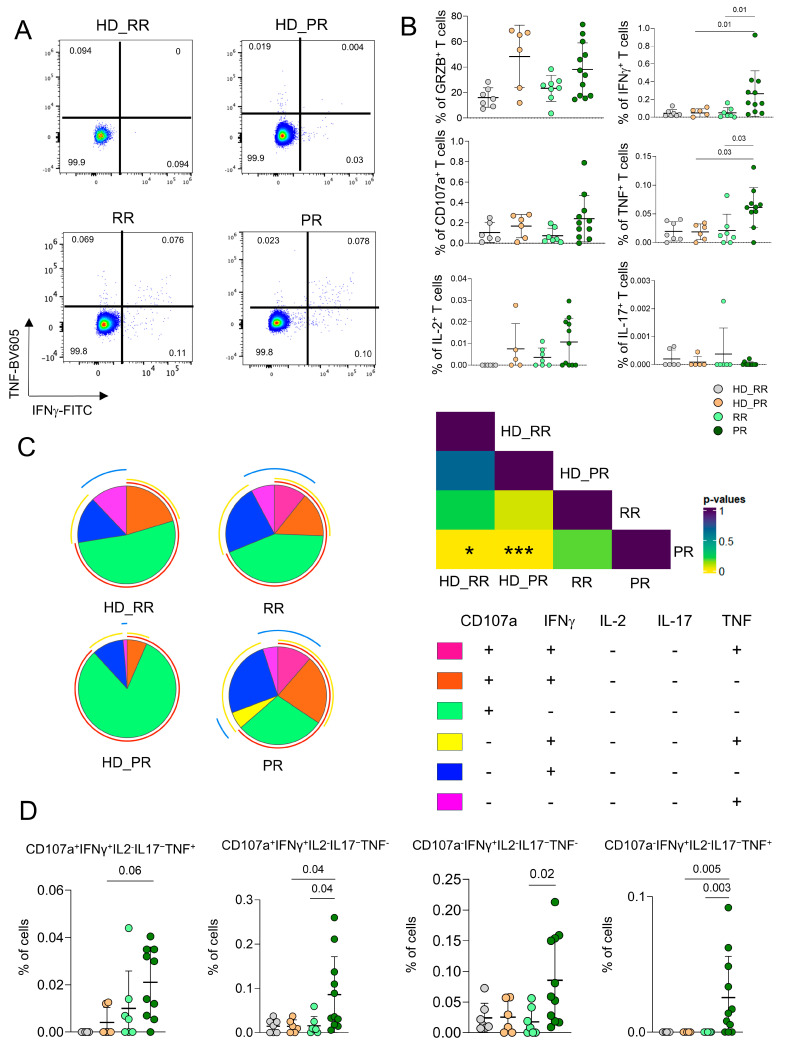
CD8^+^ Ag^+^ T polyfunctional profiles. (**A**) Dot plots obtained by the flow cytometry software Flow-Jo indicate the percentages of CD8^+^ Ag^+^ cells able to produce TNF or IFNg. (**B**) Percentage of Ag^+^ CD8^+^ T cells producing different cytokines after in vitro stimulation with SARS-CoV-2 peptides. Analysis was performed as in the Legend to Figure 1. (**C**) Profile of polyfunctional antigen-specific CD8^+^ T cells. Pie charts represent the proportion of Ag^+^ CD8^+^ T cells that contain combinations of CD107a, IL-2, IL-17, IFN-*γ*, and TNF. Each color refers to specific CD8^+^ T cell population as reported in the “polyfunctionality legend” right part of the panel. * *p* < 0.05, *** *p* < 0.001. (**D**) Differences among of representative populations of polyfunctional CD8^+^ T cells. Statistical analysis as in the Legend to Figure 1.

As for CD4^+^ Ag^+^ T cells, we measured the capability of CD8^+^ Ag^+^ T cells to produce cytokines after *in vitro* stimulation by ICS. PR patients were characterized by higher percentages of cell producing pro-inflammatory cytokines such as IFN-γ and TNF when compared to RR patients and age-matched HD (Figure 4A,B). Moreover CD8^+^ Ag^+^ T cells from PR patients were much more polyfunctional when compared to age-matched HD. In particular, the main drivers of these differences were the populations of CD8^+^ Ag^+^ T cells CD107a^+^IFN-γ^+^IL-2^−^IL-17^−^TNF^−^, and CD107a^−^IFN-γ^+^IL-2^−^IL-17^−^TNF^−^, the one able to produce CD107a^+^IFN-γ^+^IL-2^−^IL-17^−^TNF^+^ whose percentages were higher in PR patients when compared to age-matched HD (Figure 4C,D).

### 3.5. Ag^+^ B Cell Response Is Low in MS Patients Undergoing aCD20 Treatment, but It Is Still Detectable

**Figure 5:**
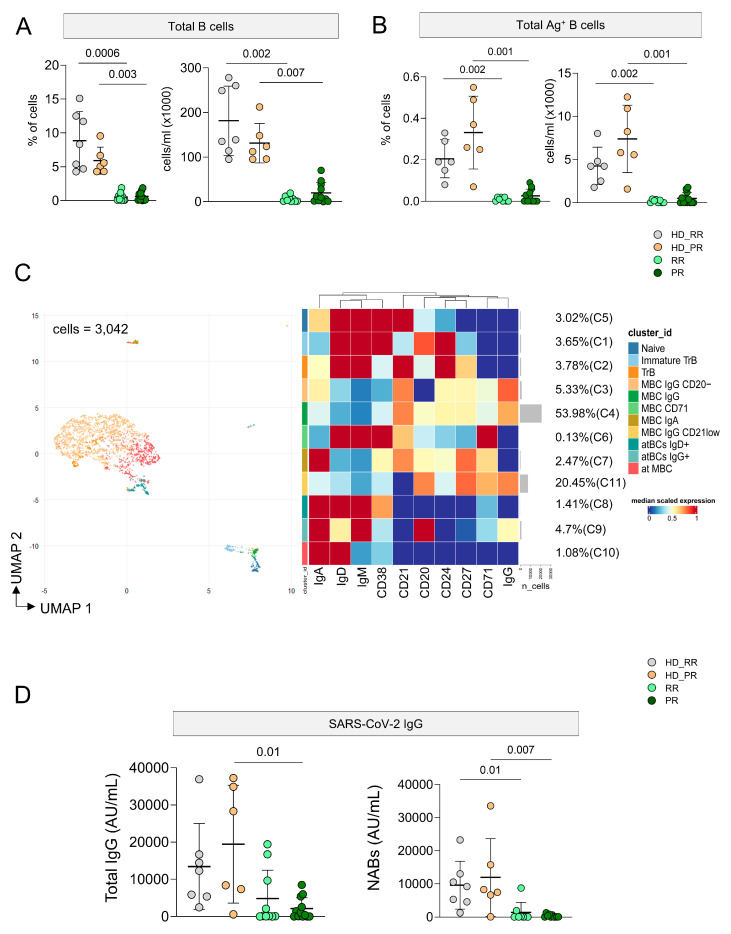
Landscape of antigen-specific B lymphocytes. (**A**) The panels with the dots report the percentage (left) or absolute number (right) of B cells. Bars indicate mean ± SD; one-sided Kruskal–Wallis test with Benjamini–Hochberg correction for multiple comparisons was used for statistical analysis, and significant adjusted q-values are reported in the figure. (**B**) Percentage and absolute number of B lymphocytes that recognize SARS-CoV-2; statistical analysis as in the previous panel. (**C**) Left: phenotype of Ag^+^ B T cells, as revealed by the Uniform Manifold Approximation and Projection (UMAP) of 3042 cells from healthy donors (HD) and MS patients treated with aCD20. Right: FlowSOM algorithm after the manual metacluster merging evidences 11 cell populations that derive from 10 lineage markers, and that are shown by the heatmap related to the median marker intensities. See Figure 1 for further details. Cell legend: N naïve, MBC memory B cells, atBC atypical memory B cells. (**D**) Anti-spike and anti-RBD IgG concentrations in plasma from HD and MS groups. Statistical analysis was performed as in previous panes: adjusted *p* values are indicated (**A**–**D**): donors and patients as in the Legend to Figure 1.

As expected, patients undergoing aCD20 displayed a lower percentage and absolute number of circulating B cells and Ag^+^ B cells when compared to HD (Figure 5A,B). However, the percentage of Ag^+^ B cells is still detectable and, for this reason, we were able to analyze the phenotype of these cells. Considering 3042 cells from MS and HD subjects we run FlowSOM (Figure 5C, Supplementary Figure S12). Eleven clusters were identified corresponding to different subpopulations of Ag^+^ B cells. 53.98% of Ag^+^ B cells were grouped into C4, a cluster that identify memory B cells (CD20^+^CD21^+^CD24^+^CD27^+^IgG^+^). A percentage of 20.45% of cells clustered into C11 that identified MBC (CD20^+^CD21^low^CD24^+^CD27^+^IgG^+^CD71^+^). A percentage of 5.33% was grouped into C3 that identified MBC (CD20^−^CD21^+^CD24^+^CD27^+^CD38^−^IgA^+^IgG^+^).

The remaining clusters are: C5 that identified naïve (CD20^+^CD21^+^CD24^+^CD27^−^IgD^+^IgM^+^); C2 transitional B cells (TrB; CD20^−^CD21^+^CD27^+^CD24^+^CD38^−^IgD^+^IgM^+^); C1 immature TrB (CD20^+^CD21^−^CD24^+^CD27^+^CD38^+^IgD^+^IgM^+^); five clusters of memory B cell MBCs defined as follows: C6 MBC CD71 (CD20^−^CD21^+^CD38^−^CD24^+^CD27^+^IgD^+^IgM^+^), C7 MBC IgA^+^(CD20^+^CD21^+^CD24^+^CD27^+^IgA^+^IgD^−^IgM^−^), C3 MBC IgG^+^ CD20^−^ (CD21^+^CD24^+^CD27^+^), and C4 MBC IgG^+^ (CD20^+^CD21^+^CD24^+^CD27^+^CD71^−^IgG^+^); C11 MBC IgG^+^ CD21 low (CD20^+^CD21^−^CD24^−^CD27^+^CD71^++^IgG^+^).

C8 atBCs IgD^+^ identified as CD21^−^CD27^−^CD20^−^IgG^−^IgD^+^; C9 atBC IgG^+^ (CD21^−^CD27^−^CD20^−^IgG^+^IgD^+^); C10 atypical memory B cells (atMBCs) as CD21^−^CD27^−^CD20^−^IgG^−^IgD^+^. Given the low number of cells, it was not possible to identify differences in different clusters among different group of patients and HD.

Plasmatic level of circulating anti-SARS-CoV-2 IgG was measured and PR patients displayed lower level of antibodies when compared to age-matched HD. Moreover, as far as the quantity of neutralizing antibodies was concerned, both group of MS patients showed lower levels if compared to age-matched HD donors (Figure 5D).

### 3.6. Principal Component Analysis Revealed That Relapsing Remitting and Progressive MS Patients Treated with Anti-CD20 Showed Similar SARS-CoV-2 Immune Features

**Figure 6:**
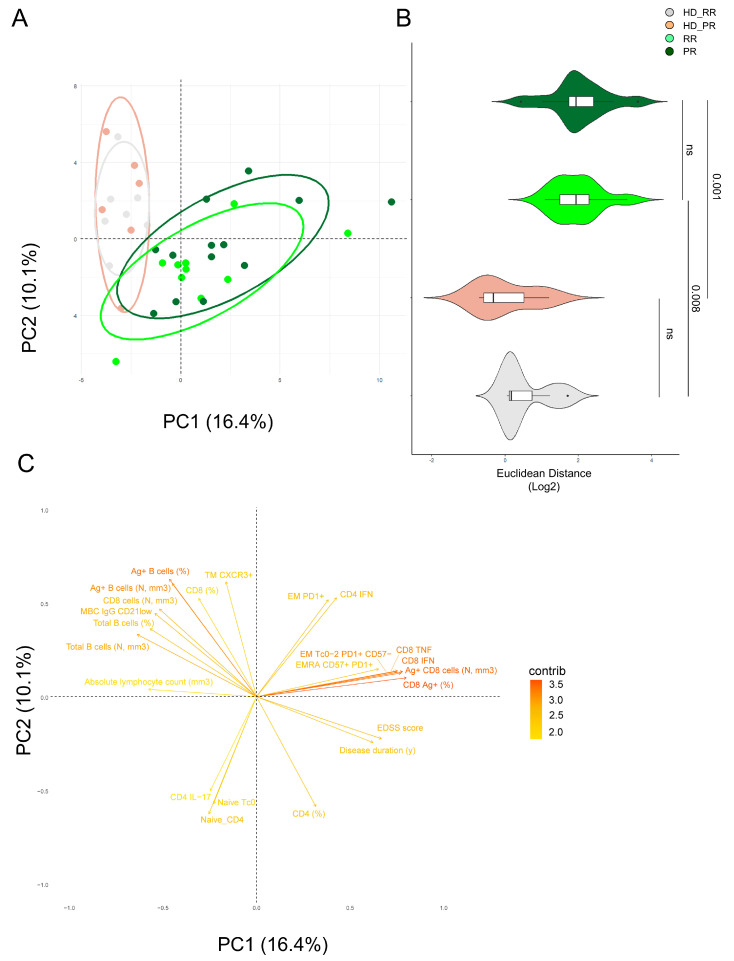
Principal component analysis of the clinical and immunological features of MS patients and healthy donors. (**A**) PCA showing the spatial distribution of vaccinated MS patients treated with aCD20 and aged-matched healthy donors (HD). (**B**) Euclidean distance to HD (together HD_RR and HD_PR, as the centroid value had the same value) has been calculated. Violin plot showing median, interquartile range (IQR) and whiskers (1.5 × IQR). Kruskal–Wallis test (one-sided) with Benjamini–Hochberg correction for multiple comparisons is used to test the differences among groups, * *p* < 0.05. (**C**) Plot displaying the variables as vector, indicating the direction of each variable to overall distribution. The strength of each variable is represented by colors: orange color represents a strong contribution; light blue color represents a milder contribution. Length and direction of the arrows indicate the weight and correlation for each parameter. Healthy donors matched with relapsing remitting (RR) patients (HD_RR): *n* = 7; healthy donors matched with progressive (PR) patients HD_PR: *n* = 6; relapsing remitting patients (RR): *n* = 10; progressive patients (PR): *n* = 12.

We used the principal component analysis (PCA) [13,22,23,31,32,34] to better summarize the results obtained so far. Based on the first two PCs (PC1 that accounted for 16.4% and PC2 that accounted for 10.1% of variance), PCA revealed that only MS patients, independently of their form of disease and age, differed for the Ag^+^ immune response when compared to age-matched HD (HD_RR vs. RR and HD-PR vs. PR), shaping two separate clusters (Figure 6A). Indeed, MS patients form a cluster on the right side of PC1 while healthy donors are on the left side. The Euclidean distance was calculated to confirm that age-matched RR HD were different from RR patients (*p* = 0.001), while age-matched PR_HD were different from PR patients (Figure 6B).

Figure 6C revealed that the main responsible of these division were, apart from clinical characteristics such as EDSS score and disease duration, the immunological features such as: percentages of Ag^+^ CD8^+^ T cells, the percentage of Ag^+^ CD8 EMRA CD57^+^PD1^+^ and the percentage of those who produced TNF and IFN-g and those with degranulation capacity (CD107a) and the percentage of Ag^+^ CD4^+^ T cells producing IFN-g. On the contrary, the main features responsible for the clusterization of HD were, apart from the percentage and absolute number of B cells and Ag^+^ B cells, the percentages of subpopulation like MBC IgG CD20^−^ and the percentages of Ag^+^ CD4^+^TM CXCR3^+^ T cells.

## 4. Discussion

The PR form of multiple sclerosis carries a higher risk of severe SARS-CoV-2 infection compared to the RR form [35]. Studies on severe COVID-19 risk in MS patients treated with anti-CD20 therapies included both RR and PR patients but used various reference groups (such as those without DMT therapy, on first-line therapies, or on other high-efficacy therapies). This variation made it unclear whether the increased risk of severe COVID-19 was due to patient factors like age, neurological disability, and comorbidities, or the immunosuppressive effects of anti-CD20 therapies (reviewed in [36]).

Thus, the aim of this study was to determine whether patients with the RR or PR forms of MS, treated with aCD20, who had been vaccinated against COVID-19 would generate a different T and B cell response against SARS-CoV-2, given the fact that their clinical background was different in terms of age and disability. Therefore, by using multiparametric flow cytometry, we carefully investigated their antigen-specific cell phenotype and function. Overall, we found that PR MS patients develop an Ag^+^ T cells response which is characterized by higher percentage of Ag^+^ T cells producing CD107a, a higher percentage of cells CD8^+^ Ag^+^ T cells, a higher percentage of Th1 Ag^+^ CD8^+^ T cells and lower plasmatic levels of SARS-CoV-2 IgG when compared to RR MS patients and age-matched healthy donors.

We and others previously reported that the immune system of MS patients displays different characteristic of senescence, such as higher percentages of terminally differentiated T cells expressing CD57 and PD-1 [21,37,38,39,40,41,42,43,44,45]. Moreover, different DMT dampen these characteristics [20], as far as the antigen specific response to SARS-CoV-2 after vaccination was evaluated. Indeed, RR patients treated with fingolimod, natalizumab, and aCD20 therapies display a different scenario of immune correlate of protection to SARS-CoV-2.

Here, we found that PR patients displayed high percentages of Ag^+^ CD4^+^ T cells producing CD107a, a population of cells that differentiate in an environment rich of senescent cells, and that decreasing the number of senescent cells with chemical senolytic drugs was enough to stop this differentiation [46]. Indispensable for the development of cytotoxic CD4 cells are CD4^+^ T cell effectors that have to simultaneously recognize local antigens and encounter IL-15 induced by type I IFN [47]. Additionally, in hospitalized COVID-19 patients with weakened humoral responses, an increase in T cells with cytotoxicity-associated transcripts was noted, indicating that these cells may contribute to the depletion of germinal center B cells seen in severe SARS-CoV-2 cases [48]. Interestingly, a high amount of this cell type is a signature of the supercentenarians’ immune system. These cells are accumulated through massive clonal expansion, with the most frequent clonotypes accounting for 15 to 35% of the entire CD4^+^ T cell population, which also shares a transcriptome that is nearly identical to that of cytotoxic CD8^+^ T cells. This indicates that these cells utilize a similar transcriptional program [49], and could indirectly suggest that PR patients, who are older than RR, could maintain a sustained and efficient immune response to SARS-CoV-2 breakthrough infection.

As far as the population of CD8^+^ Ag^+^ T cells is concerned, we have seen that PR patients develop higher percentages of CD8^+^ Ag^+^ T cells and very low levels of both anti-IgG SARS-CoV-2 and anti-RBD antibodies. A similar observation was reported in a cohort of MS patients treated with aCD20 (both PR and RR form) [27]. Furthermore, it has been shown that CD8^+^ T cells are rapidly mobilized and stably maintained after prime vaccination with BNT162b2, preceding the maturation of other immune responses [50]. Additionally, a study examining the impact of SARS-CoV-2 variants on total CD4^+^ and CD8^+^ T cell reactivity in infected or vaccinated individuals indicates that total T cell reactivity remains largely intact despite some variations [51]. A final, non-mutually exclusive possibility is that antibodies or immune complexes could block the inhibitory Fc receptor FcγRIIB on dendritic cells or CD8^+^ T cells. This could be due to the fact that the insufficient clearance of the virus by vaccine-induced antibodies could drive CD8^+^ T cell activation and proliferation, or that the pool of regulatory B cells, i.e., those B cells producing IL-10 [52,53], might directly attenuate CD8^+^ T cell responses. A final, non-mutually exclusive possibility is that antibodies or immune complexes could block the inhibitory Fc receptor FcγRIIB on dendritic cells or CD8^+^ T cells.

Even if the number of patients and age- and sex-matched healthy donors enrolled in the study is relatively small, we underline that the timing after the third vaccination is matched and we evaluated a long-term immune response after vaccination (6 months), indeed before the already observed decrease in protection [54]. Moreover, the extensive longitudinal analysis from Apostolidis already evaluated the early immune response of anti-CD20-treated patients before the first vaccine dose, 10–12 days after the first vaccine dose, immediately before the second vaccine dose, 10–12 days after the second vaccine dose and 25–30 days after the second vaccine dose pointing out that since the first vaccination these patients are not able to develop an antigen-specific B cells response if compared to healthy donors and that the second booster increased the percentage of antigen-specific T cell response [27]. In conclusion, it is reasonable to hypothesize that, overall, MS patients and healthy donors are characterized by different immunological features that are mainly due to the disease and to the use of aCD20, considering the lower percentages and absolute number of B cells. In any case, we found that drug-induced B cell inhibition did not influence T cell responses, which could be central to SARS-CoV-2 elimination in case of a breakthrough infection.

## 5. Conclusions

Our study demonstrates that PR MS patients exhibit higher percentages of antigen-specific CD8^+^ T cells and CD107a-producing T cells, suggesting that enhanced cytotoxic activity may compensate for their deficit in antibody production. Indeed, these patients show lower anti-SARS-CoV-2 IgG levels compared to RR MS patients and healthy donors. This finding indicates that in PR MS patients, the control of SARS-CoV-2 infection may rely more on T cell-mediated immunity than on antibody responses.

Overall, our findings reveal that immune responses in MS patients vary with disease severity and the type of MS therapy administered. Specifically, PR MS patients, who typically present with more severe disease than RR MS patients, display distinct immune profiles. This highlights the need to consider both the form of MS and the therapeutic regimen when evaluating COVID-19 vaccine responses and, in general, when developing immunization strategies.

## Figures and Tables

**Table 1:** Patient demographic and clinical characteristics table.

Study Population (*N* = 36)	HD_RR (*N* = 7)	HD_PR (*N* = 6)	RR (*N* = 10)	PR (*N* = 13)
Age, median (IQR), y	35.0 (29.0–42.5)	57.5 (55.5–66.25)	35.5 (30.25–38.0)	57.0 (53.0–61.0)
Females, *n* (%)	6.0 (85.7)	4.0 (66.7)	6.0 (60.0)	10.0 (76.9)
Disease duration, median (IQR), y	─	─	11.5 (10.0–12.75)	17.0 (10.0–24.00)
Disability by EDSS score, median (IQR)	─	─	2.75 (1.25–4.38)	6.0 (4.00–6.50)
Time from last treatment starts, median (IQR), y	─	─	0.526 (0.50–0.57)	0.528 (0.50–0.60)
Time from last infusion to last vaccination, median (IQR), months	─	─	3.70 (0.19–4.81)	3.41 (1.44–4.33)
				
Absolute lymphocyte count, median (IQR), 10^3^cells/mm^3^	2.22 (1.95–2.33)	2.225 (2.23–2.23)	1.61 (1.38–2.06)	1.74 (1.49–2.22)
CD19 B─cell count, median (IQR), *n*/mm^3^	138,944 (124,511–254,738)	118,148 (99,847–142,122)	4381 (1010–11,212)	7822 (1125–30,770)
				
Breakthrough COVID─19 after full vaccination, *n* (%)	2.00 (66.70)	NA	1.00 (10.00)	2.00 (15.40)
SARS─CoV─2 IgGII titer after full vaccination, AU/mL				
Median (IQR)	12,288.50 (5334–16,687.50)	28,306.60 (7348.60–34,873.30)	135.65 (41.80–9656)	1089.1 (9.25–7176.0)
SARS─CoV─2 RBD IgG titer after full vaccination, AU/mL				
Median (IQR)	9167.221 (4539.23–13,011.16)	7829.5923 (6621.08–15,762.73)	83.02 (4.80–8700.06)	346.13 (0.41–3760.41)
Time from first vaccination to sampling, median (IQR), months	14.25 (9.10–16.56)	10.71 (7.26–12.83)	11.67 (9.52–15.40)	10.94 (9.63–15.01)
Time from last vaccination to sampling, median (IQR), months	3.61 (1.74–6.60)	3.28 (0.92–6.04)	2.56 (1.695–8.22)	2.96 (2.20–7.705)
Type of third vaccine dose				
BNT162b2, *n* (%)	5 (71.43%)	3 (50.00%)	8 (80.00%)	9 (69.23%)
mRNA─1273, *n* (%)	2 (28.57%)	3 (50.00%)	2 (20.00%)	4 (30.77%)

## Data Availability

All data generated or analysed in this study are included in this published article (and its supplementary information files). Raw data are reported in the Supplementary Materials. Further inquiries can be directed to the corresponding authors.

## Supplementary Materials

The following supporting information can be downloaded at: https://www.mdpi.com/article/10.3390/vaccines12080924/s1, Supplementary Table S1. mAbs used in AIM assay; Supplementary Table S2. mAbs used in B cell panel; Supplementary Table S3. mAbs used in ICS protocol; Supplementary Figure S1. Gating strategy for the identification and characterization of antigen-specific CD4^+^ T cells (AIM assay); Supplementary Figure S2. Gating strategy for the identification and characterization of antigen-specific CD4^+^ T cells (AIM assay); Supplementary Figure S3. Gating strategy and representative plots of intracellular staining analysis of cytokine producing cells (ICS) after overnight stimulation with spike protein compared to unstimulated control; Supplementary Figure S4. Gating strategy before computational analysis of Ag^+^ B cells; Supplementary Figure S5. Uniform Manifold Approximation and Projection (UMAP) plot shows the 2D spatial distribution of CD4^+^ cells from 13 heallthy donors vaccinated against SARS-CoV2 and 23 patients with relapsing-remitting or progressive relapsing multiple sclerosis and vaccinated against COVID-19; Supplementary Figure S6. Uniform Manifold Approximation and Projection (UMAP) plot shows the 2D spatial distribution of cells from 13 heallthy donors vaccinated against SARS-CoV2 and 23 patients with relapsing-remitting or progressive relapsing multiple sclerosis and vaccinated against COVID-19; Supplementary Figure S7. Detailed statistical analysis of CD4+clusters obtained using FlowSOM. On the left, dot plots show the percentage of cells in different groups of MS patients (PR and RR) and healthy donors (HD-RR and HD-PR); Supplementary Figure S8. Uniform Manifold Approximation and Projection (UMAP) plot shows the 2D spatial distribution of CD8^+^ cells from 13 heallthy donors vaccinated against SARS-CoV2 and 23 patients with relapsing-remitting or progressive relapsing multiple sclerosis and vaccinated against COVID-19; Supplementary Figure S9. Uniform Manifold Approximation and Projection (UMAP) plot shows the 2D spatial distribution of CD8 cells from 13 heallthy donors vaccinated against SARS-CoV2 and 23 patients with relapsing-remitting or progressive relapsing multiple sclerosis and vaccinated against COVID-19; Supplementary Figure S10. Detailed statistical analysis of CD8+clusters obtained using FlowSOM. On the left, dot plots show the percentage of cells in different groups of MS patients (PR and RR) and healthy donors (HD-RR and HD-PR); Supplementary Figure S11. Uniform Manifold Approximation and Projection (UMAP) plot shows the 2D spatial distribution of B cells from 13 heallthy donors vaccinated against SARS-CoV2 and 23 patients with relapsing-remitting or progressive relapsing multiple sclerosis and vaccinated against COVID-19; Supplementary Figure S12. Uniform Manifold Approximation and Projection (UMAP) plot shows the 2D spatial distribution of B cells from 13 heallthy donors vaccinated against SARS-CoV2 and 23 patients with relapsing-remitting or progressive relapsing multiple sclerosis and vaccinated against COVID-19; Source Data File.
