# Supplementary material for: SARS-CoV-2 Vaccination Responses in Anti-CD20-Treated Progressive Multiple Sclerosis Patients Show Immunosenescence in Antigen-Specific B and T Cells
Source: Vaccines (Basel). 2024 Aug 17;12(8):924. doi: 10.3390/vaccines12080924 (PMC11360119; doi:10.3390/vaccines12080924)
Supplement: Supplementary file 1 [file vaccines-12-00924-s001.zip › vaccines-3110172 - Supplementary Materials Final.pdf]

# Supplementary Information

**Supplementary Table S1**. mAbs used in AIM assay.

| **Target** | **Dye** | **Clone** | **Producer** | **Catalog number** | **Lot number** | **Titer (uL)/100 uL** |
|---|---|---|---|---|---|---|
| **PromoFluor840** | Maleimide | N/A | Promocell | PK-PF840-3- 01 | | 0.3 |
| **CD45RA** | FITC | 2H4 | Beckman Coulter (DuraClone IM T) | B53328 | | - |
| **CCR7** | PE | G043H7 | Beckman Coulter (DuraClone IM T) | B53328 | | - |
| **CD28** | ECD | CD28.2 | Beckman Coulter (DuraClone IM T) | B53328 | | - |
| **PD-1** | PC5.5 | PD1.3.5 | Beckman Coulter (DuraClone IM T) | B53328 | | - |
| **CD27** | PC7 | 1A4.CD27 | Beckman Coulter (DuraClone IM T) | B53328 | | - |
| **CD4** | APC | 13B8.2 | Beckman Coulter (DuraClone IM T) | B53328 | | - |
| **CD8** | A700 | B9.11 | Beckman Coulter (DuraClone IM T) | B53328 | | - |
| **CD3** | APC-A750 | UCHT-1 | Beckman Coulter (DuraClone IM T) | B53328 | | - |
| **CD57** | Pacific Blue | NC1 | Beckman Coulter (DuraClone IM T) | B53328 | | - |
| **CD45** | Krome Orange | J33 | Beckman Coulter (DuraClone IM T) | B53328 | | - |
| **CXCR3** | BV785 | G025H7 | BioLegend | 353738 | B302668 | 1.25 |
| **CCR6** | BUV496 | 11A9 | Becton Dickinson | 612948 | 1114714 | 1.25 |
| **CXCR5** | BUV661 | RF8B2 | Becton Dickinson | 741559 | 1298915 | 0.6 |
| **CD69** | BV650 | FN50 | BioLegend | 310934 | B346313 | 2.5 |
| **CD137** | BUV395 | 4B4-1 | Becton Dickinson | 745737 | 1298922 | 1.25 |
| **CD95** | BV605 | DX2 | BioLegend | 305628 | B344380 | 2.5 |

**Supplementary Table S2**. mAbs used in B cell panel.

| Target | Dye | Clone | Producer | Catalog Number | Lot Number | Titer (uL)/100uL |
|---|---|---|---|---|---|---|
| **PromoFluor840** | Maleimide | N/A | Promocell | PK-PF840-3-01 | | 0.3 |
| **CD45** | Krome Orange | J33 | Beckman Coulter (DuraClone IM B) | B53318 | | - |
| **CD19** | ECD | J3-119 | Beckman Coulter (DuraClone IM B) | B53318 | | - |
| **CD21** | PE | BL13 | Beckman Coulter (DuraClone IM B) | B53318 | | - |
| **CD27** | PC7 | 1A4CD27 | Beckman Coulter (DuraClone IM B) | B53318 | | - |
| **CD24** | APC | ALB9 | Beckman Coulter (DuraClone IM B) | B53318 | | - |
| **CD38** | APC-A750 | LS198-4-3 | Beckman Coulter (DuraClone IM B) | B53318 | | - |
| **IgD** | FITC | IA6-2 | Beckman Coulter (DuraClone IM B) | B53318 | | - |
| **IgM** | Pacific Blue | SA-DA4 | Beckman Coulter (DuraClone IM B) | B53318 | | - |
| **Streptavidin** | BV650 | - | BioLegend | 405231 | B347044 | 0.3 |
| **Streptavidin** | BUV661 | - | Becton Dickinson | 612979 | 1188291 | 0.3 |
| **Streptavidin** | AF700 | - | ThermoFisher | S21383 | 2286302 | 0.1 |
| **S-protein** | Biotin | - | R&D | BT10549 | DOJH0421071 | 4.5 |
| **CD20** | BV785 | 2H7 | BioLegend | 302356 | B337363 | 0.6 |
| **CD71** | BUV395 | M-A712 | Becton Dickinson | 743308 | 1341511 | 1.25 |
| **IgG** | BUV496 | G18-154 | Becton Dickinson | 741172 | 1341490 | 1.25 |
| **IgA** | PerCP-Vio700 | 1S11-8E10 | Miltenyi Biotec | 130-113-478 | 5211109889 | 0.5 |

**Supplementary Table S3**. mAbs used in ICS protocol

| Target | Dye | Clone | Producer | Catalog number | Lot number | Titer (ul)/100ul |
|---|---|---|---|---|---|---|
| **LIVE/DEAD** | AQUA | N/A | ThermoFisher | L34966 | 2268307 | 1.25 |
| **CD4** | AF700 | RPA-T4 | Biolegend | 300526 | B336913 | 0.6 |
| **CD8** | APC-Cy7 | RPA-T8 | Biolegend | 301016 | B300873 | 0.6 |
| **CD3** | PE-Cy5 | UCHT1 | Biolegend | 301016 | B300873 | 0.6 |
| **IFNg** | FITC | B27 | Biolegend | 506504 | B286029 | 2.5 |
| **TNF** | BV605 | MAb11 | Biolegend | 502936 | B327946 | 3.75 |
| **IL-2** | APC | MQ1-17H12 | Biolegend | 500310 | B313276 | 2.5 |
| **IL-17a** | PE-Cy7 | BL168 | Biolegend | 512315 | B325831 | 3.75 |
| **GRZB** | BV421 | QA18A28 | Biolegend | 396414 | B311965 | 2.5 |
| **CD107a** | PE | H4A3 | Biolegend | 328608 | B321484 | 0.3 |

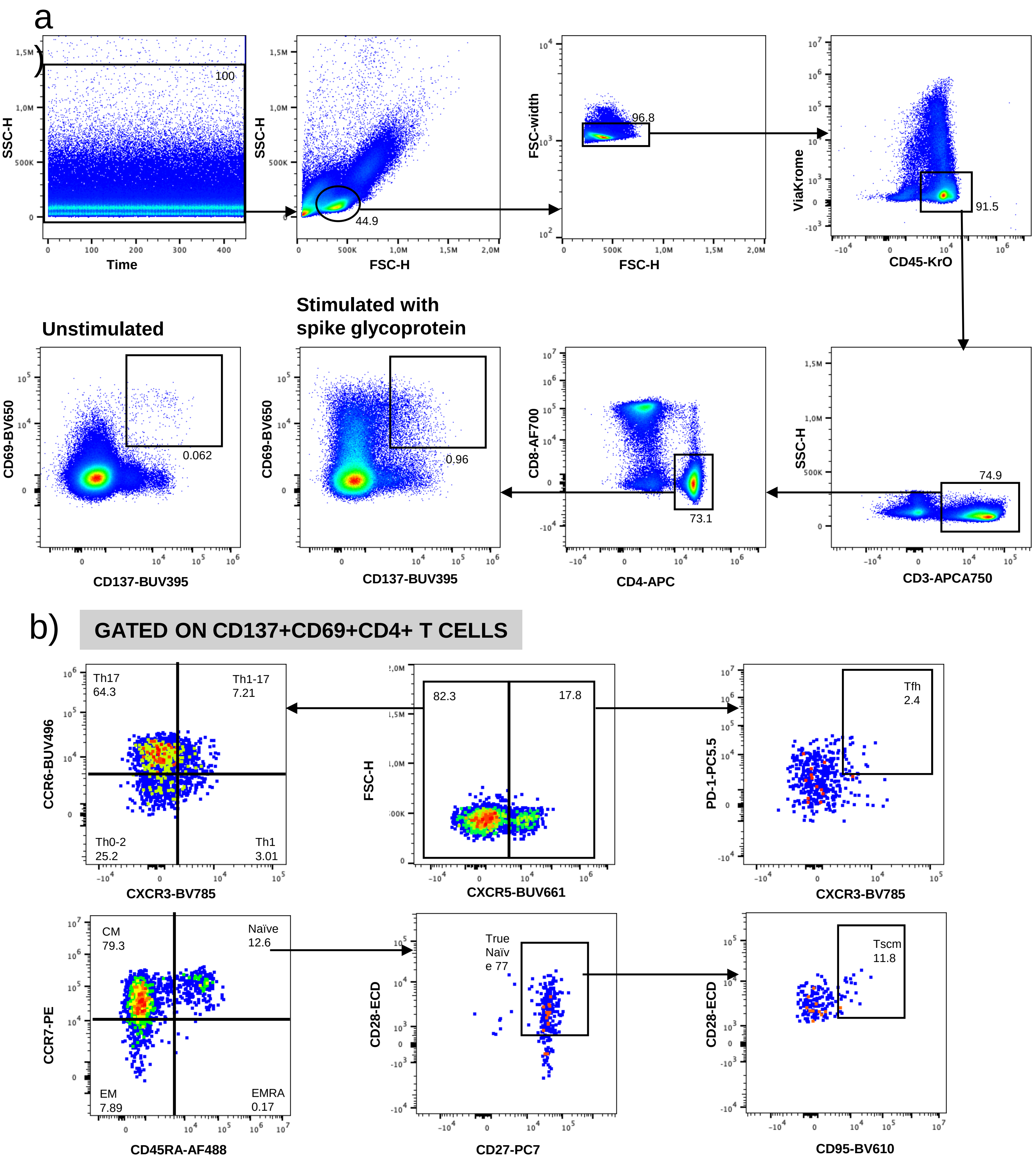


**Supplementary Figure S1.** Gating strategy for the identification and characterization of antigen-specific CD4⁺ T cells (AIM assay). (a) On a bivariate plot of Time vs. SSC-H, create and place a rectangular region to include all valid events acquired in chronologic homogeneity and avoid fluidic perturbances. Forward and side scatter (FSC and SSC) gating is used to identify cells of interest based on the relative size and complexity of the cells, while removing debris and other events that are not of interest. Further gating is done in an FSC-H and FSC-Width dot plot to eliminate doublets. On a bivariate plot of CD45 vs. ViaKrome (viability) select $CD45^+$, $ViaKrome^-$ cells (viable cells). On a bivariate plot of CD3 vs SSC-H select CD3 T lymphocytes. Select $CD4^+$ T cells and evaluate $CD69^+$ $CD137^+$ Antigen Specific T cells. b) Gating strategy to identify and characterize T helper (Th), circulating T follicular helper (cTFH), Naïve (N), Central memory (CM), effector memory (EM), terminally differentiated effector memory (EMRA), true naive and TSCM among $Ag^+CD4^+$ T cells.

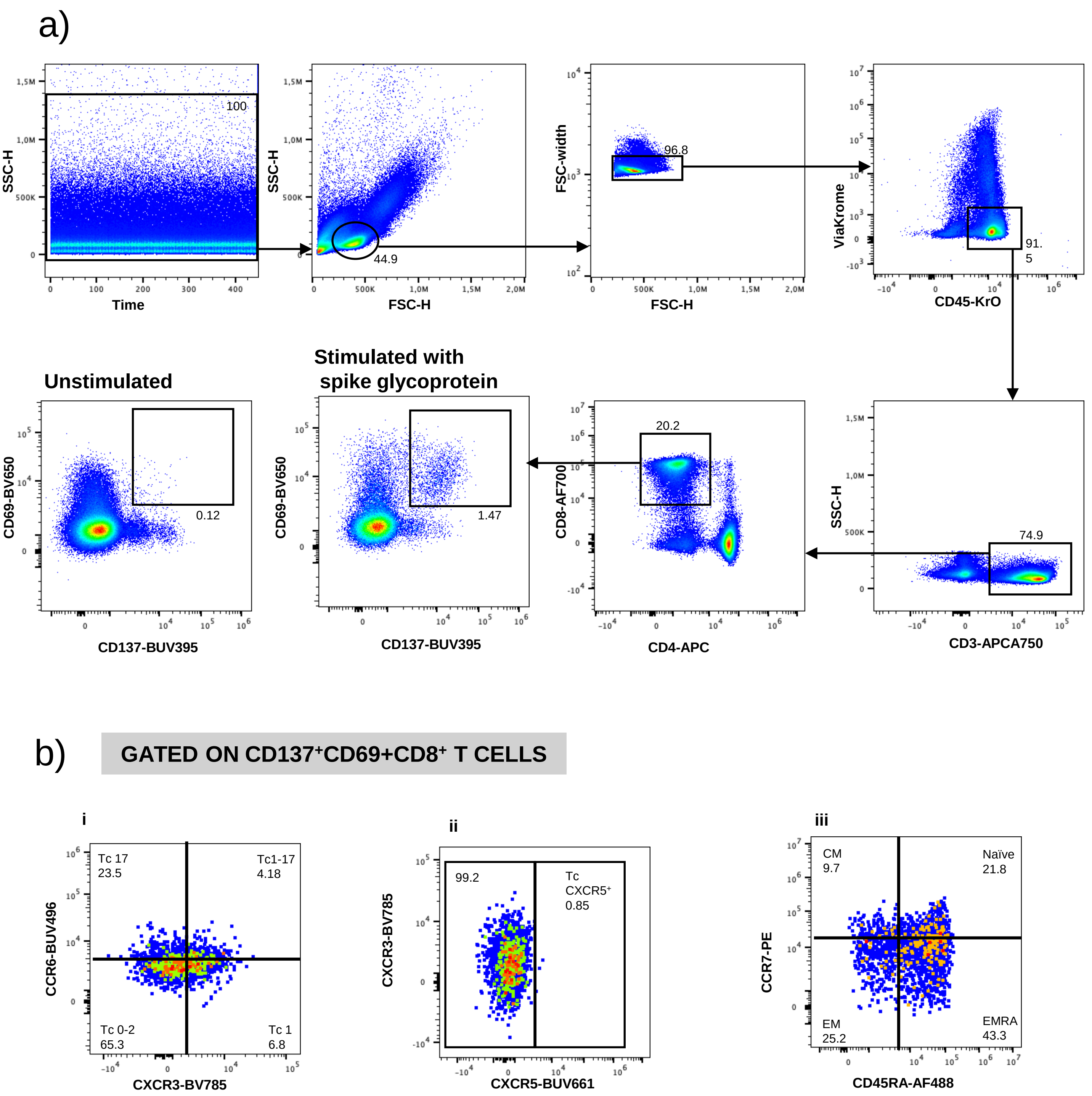


**Supplementary Figure S2.** Gating strategy for the identification and characterization of antigen-specific $CD4^+$ T cells (AIM assay). (a) On a bivariate plot of Time vs. SSC-H, create and place a rectangular region to include all valid events acquired in chronologic homogeneity and avoid fluidic perturbances. Forward and side scatter (FSC and SSC) gating is used to identify cells of interest based on the relative size and complexity of the cells, while removing debris and other events that are not of interest. Further gating is done in an FSC-H and FSC-Width dot plot to eliminate doublets. On a bivariate plot of CD45 vs. ViaKrome (viability) select $CD45^+$, $ViaKrome^-$ cells (viable cells). On a bivariate plot of CD3 vs SSC-H select CD3 T lymphocytes. Select $CD8^+$ T cells and evaluate $CD69^+$ $CD137^+$ Antigen Specific T cells. (b) Gating strategy to identify and characterize i) T cyotoxic (Tc), ii)Tc $CXCR5^+$,iii) Naïve (N), central memory (CM), effector memory (EM) and terminally differentiated effector memory (EMRA) among $Ag^+CD8^+$ T cell populations.

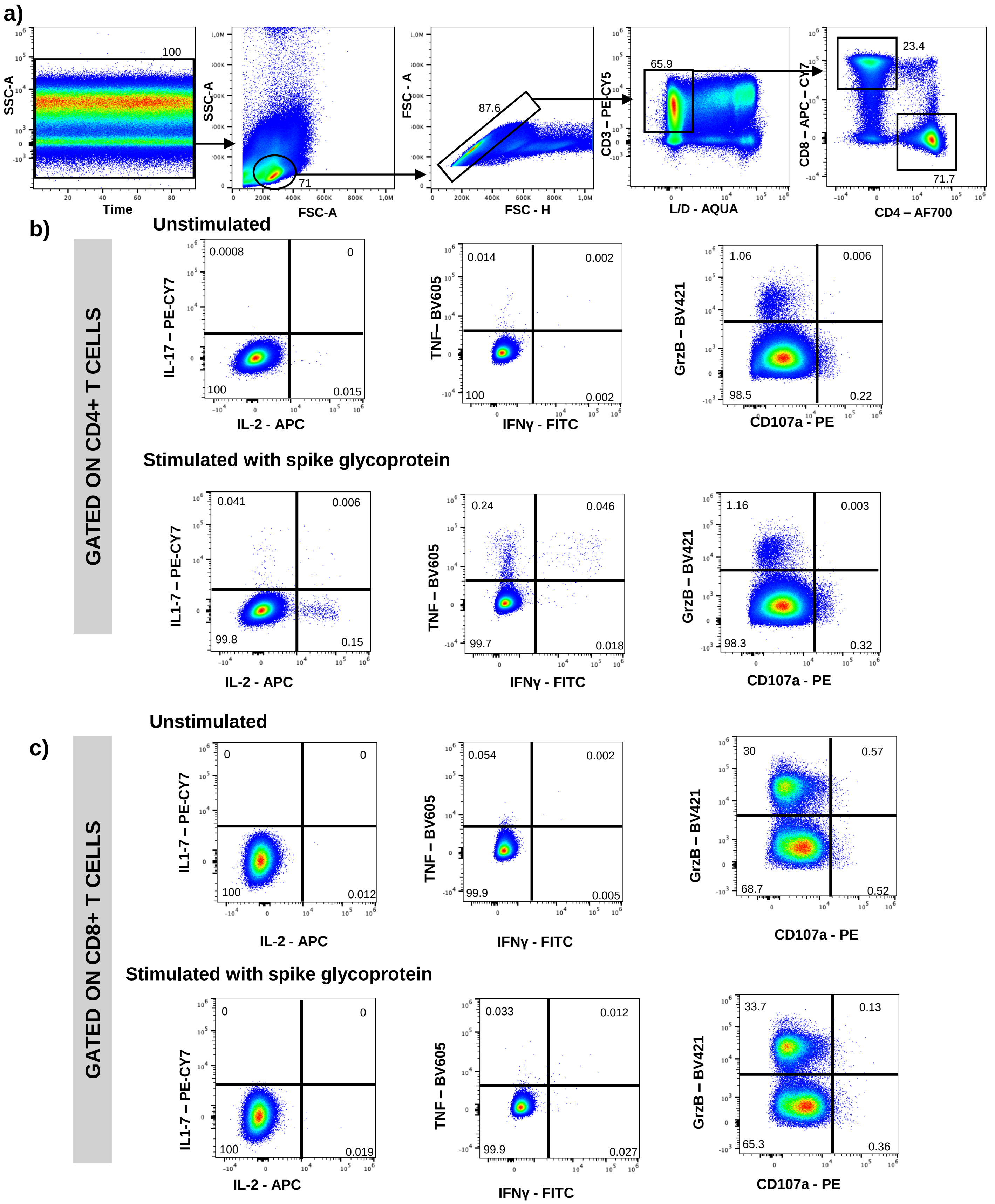


**Supplementary Figure S3.** Gating strategy and representative plots of intracellular staining analysis of cytokine producing cells (ICS) after overnight stimulation with spike protein compared to unstimulated control. Cytokine production and polyfunctionality of antigen-specific $CD4^+$ T cells (panel b) and $CD8^+$ T cells (panel c) . Numbers in the dot plots indicate the percentage of $CD4^+$ and $CD8^+$ cells identified by the gates. Comparison between the total production of IFNg, TNF, IL-17, IL-2, CD107a, and GZMB .

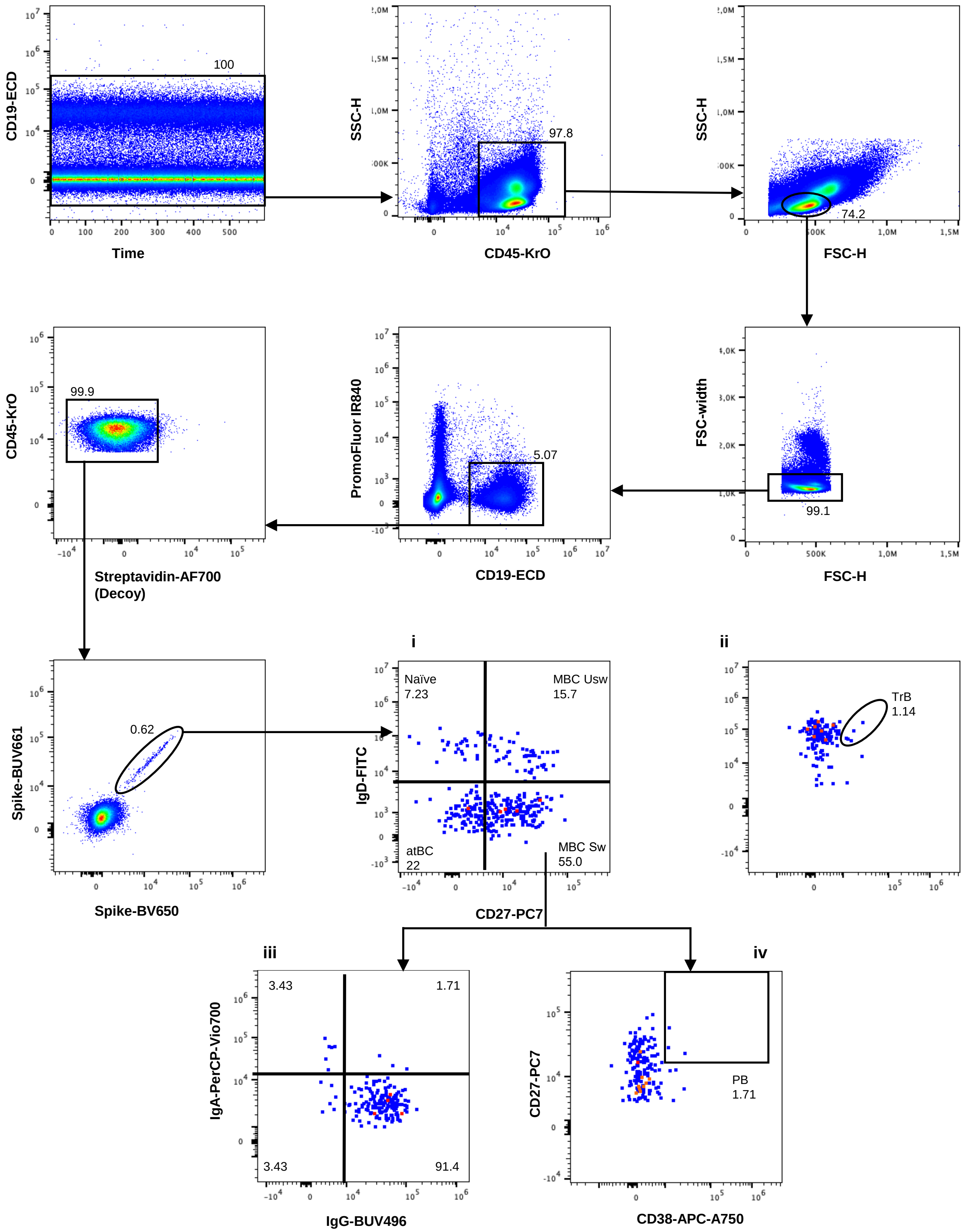


**Supplementary Figure S4.** Gating strategy before computational analysis of Ag+ B cells. The Time *vs* CD19-ECD gate was used to exclude unstable flow during acquisition. Leukocytes were selected as CD45+ cells. Next, lymphocytes were selected based on their physical characteristics, and doublets were excluded from the analysis by utilizing forward scatter height (FSC-H) and forward scatter width (FSC-W) parameters. Living B cells were selected as PromoFluor— and CD19+ . Ag+ (Spike-BUV661+ and Spike-BV650+) and Ag− ((Spike-BUV661− and Spike-BV650−) B cells were displayed. Inside Ag+ B cells. we identified **i)** Naïve cells, Memory Unswitched B cells (MBC Usw), Memory Switched B cells (MBC Sw) and atypical B cells (atBC); ii) transitional B cells (TrB). Within MBC Sw gate. iii) the quadrant plots reported the different percentages of IgA+. IgG+ cells while iv) the dot plots plasmablast (PB) percentage.

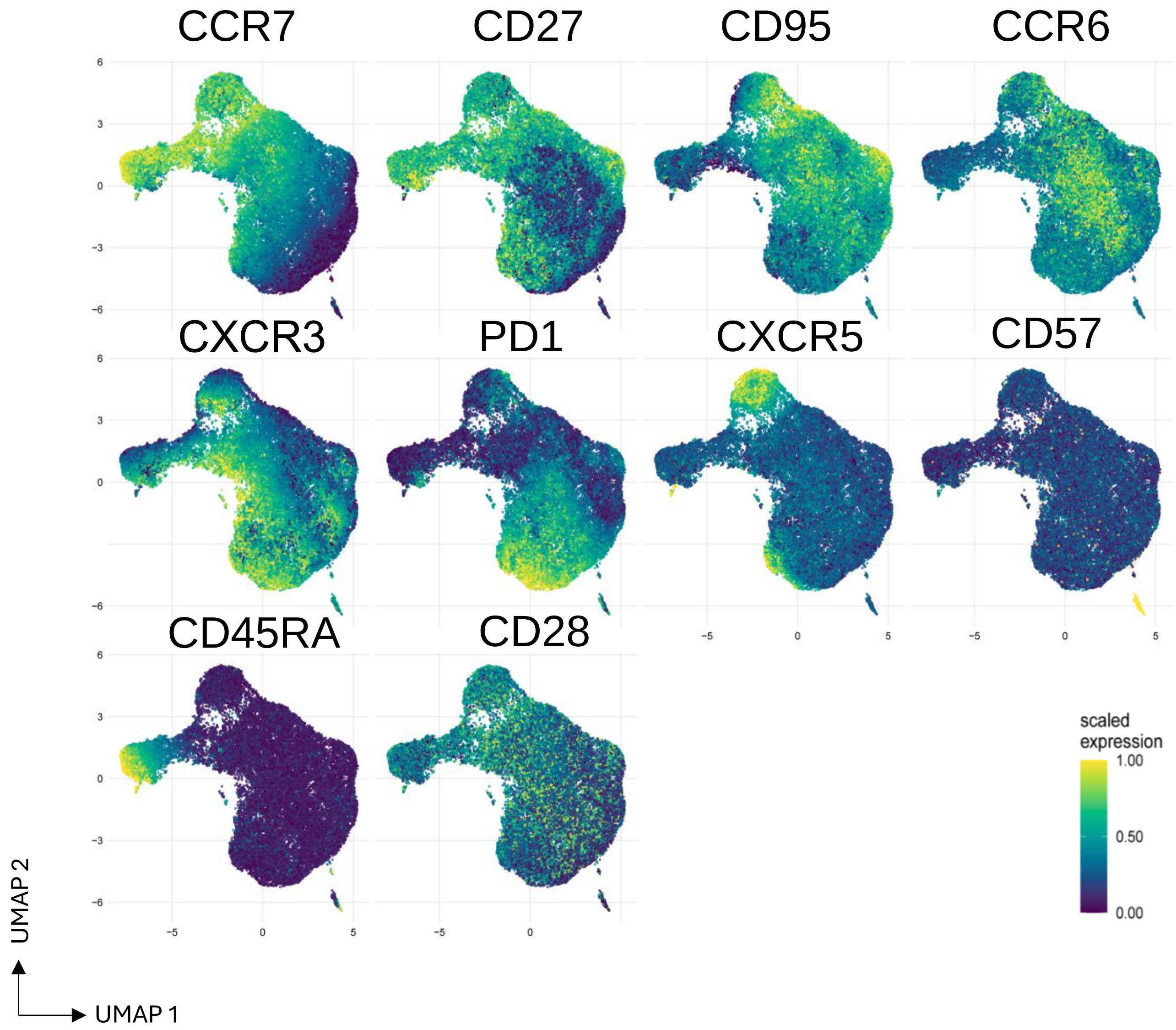


**Supplementary Figure S5.** Uniform Manifold Approximation and Projection (UMAP) plot shows the 2D spatial distribution of CD4⁺ cells from 13 heallthy donors vaccinated against SARS-CoV2 and 23 patients with relapsing-remitting or progressive relapsing multiple sclerosis and vaccinated against COVID-19. UMAP graphs colored by the expression of 10 markers used for CD4⁺ antigen specific T cell phenotyping. Blue represents lower expression while yellow represent higher expression.

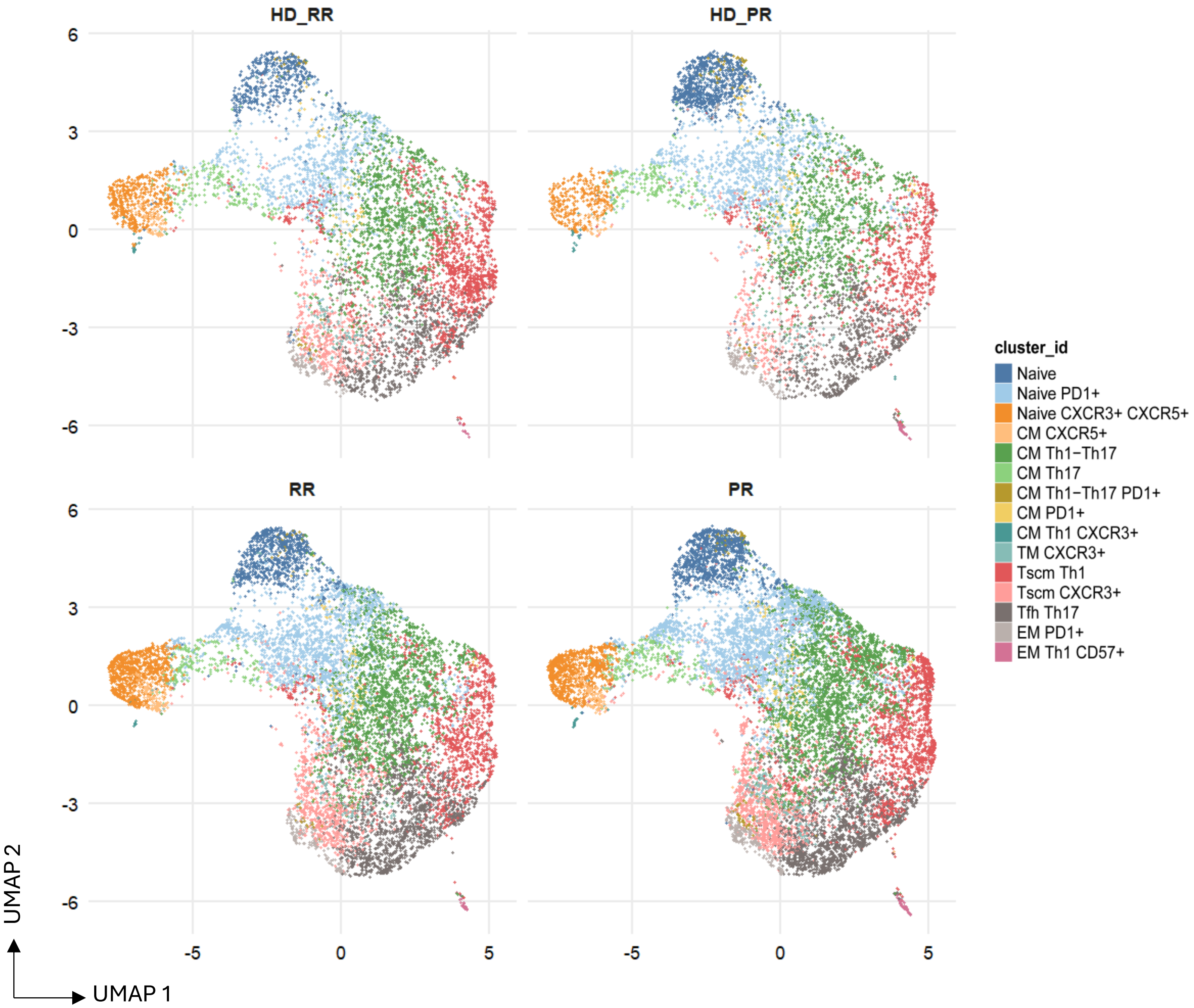


**Supplementary Figure S6.** Uniform Manifold Approximation and Projection (UMAP) plot shows the 2D spatial distribution of cells from 13 heallthy donors vaccinated against SARS-CoV2 and 23 patients with relapsing-remitting or progressive relapsing multiple sclerosis and vaccinated against COVID-19. UMAP graphs stratified by group**:** HD-PR: healthy donors-progressive relapsing (N=6); HD-RR: healthy donors-relapsing-remitting (N=7); PR: progressive relapsing (N=13); RR: relapsing-remitting(N=10).

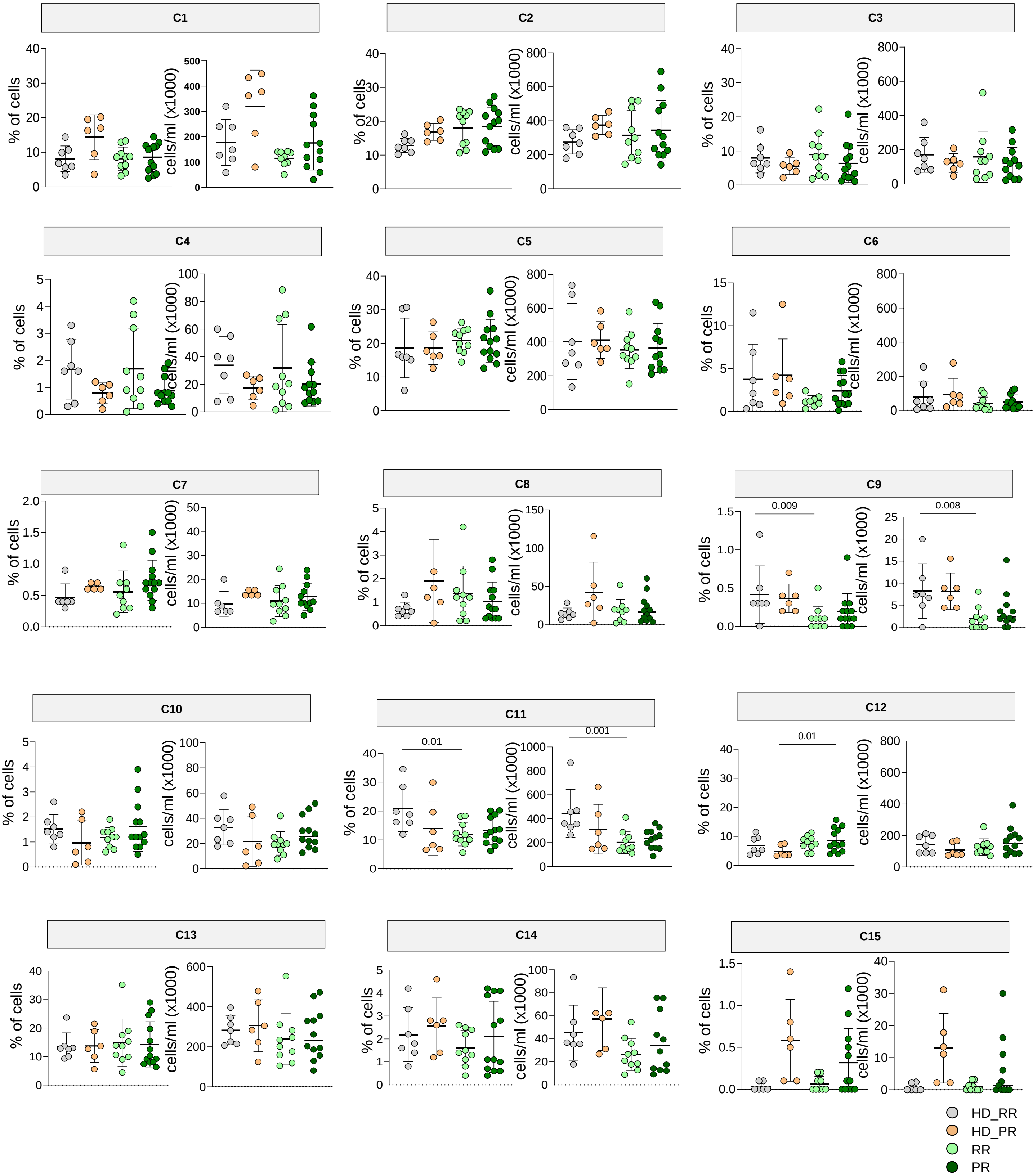


**Supplementary Figure S7.** Detailed statistical analysis of CD4⁺clusters obtained using FlowSOM. On the left, dot plots show the percentage of cells in different groups of MS patients (PR and RR) and healthy donors (HD-RR and HD-PR). On the right, dot plots show the absolute number of cells in different groups of patients and HD. Scatter plots show individual values; the central bar represents the mean ± SD. Kruskal–Wallis test (one-sided) with Benjamini–Hochberg correction for multiple comparisons. Tables display statistically significant q-value and individual p-value obtained. For all graphs: HD-PR: healthy donors-progressive relapsing (N=6); HD-RR: healy donors-relapsing-remitting (N=7); PR: progressive relapsing (N=13); RR: relapsing-remitting(N=10).

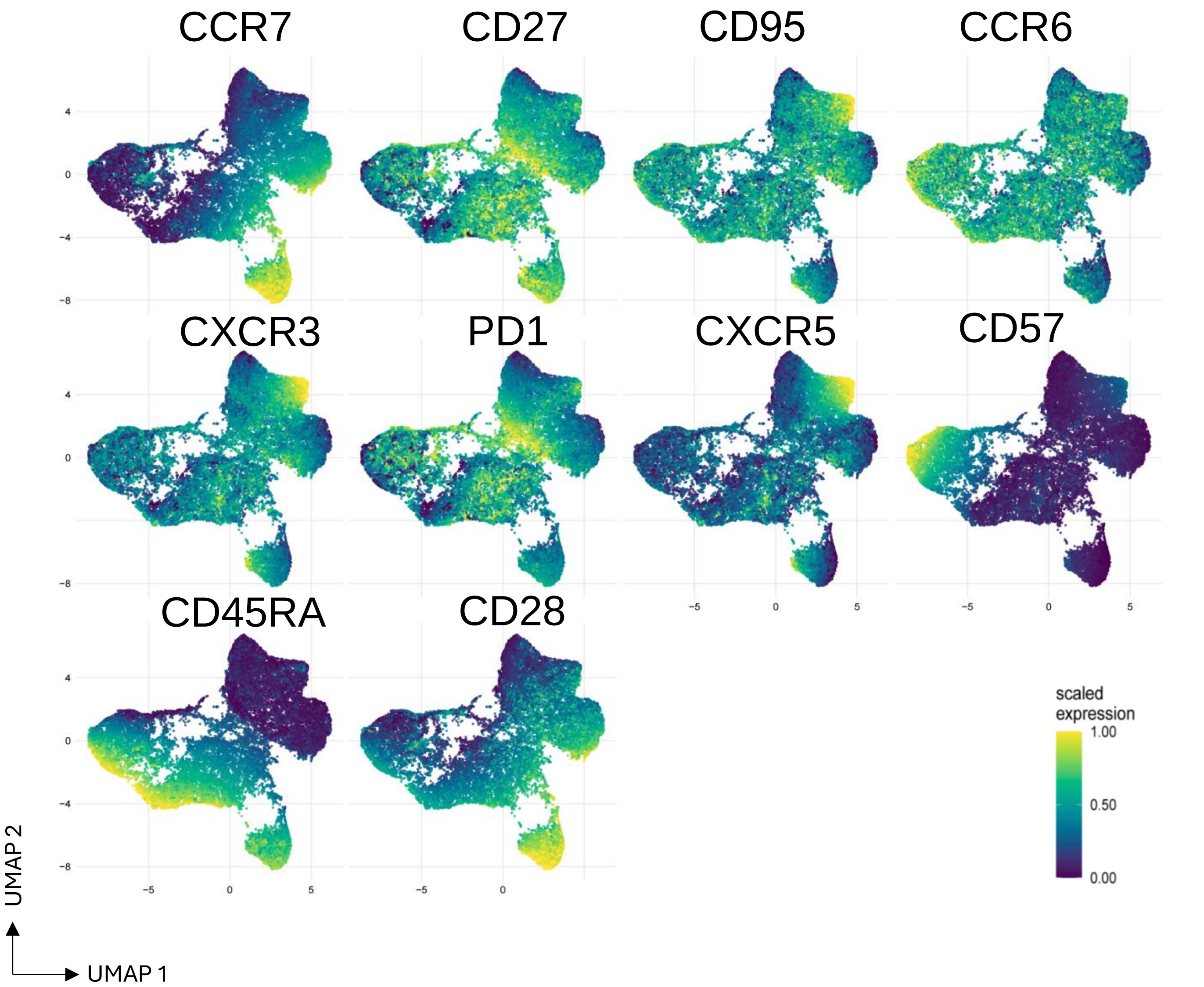


**Supplementary Figure S8.** Uniform Manifold Approximation and Projection (UMAP) plot shows the 2D spatial distribution of $CD8^{+}$ cells from 13 heallthy donors vaccinated against SARS-CoV2 and 23 patients with relapsing-remitting or progressive relapsing multiple sclerosis and vaccinated against COVID-19. UMAP graphs colored by the expression of 10 markers used for $CD8^{+}$ antigen specific T cell phenotyping. Blue represents lower expression while yellow represent higher expression.

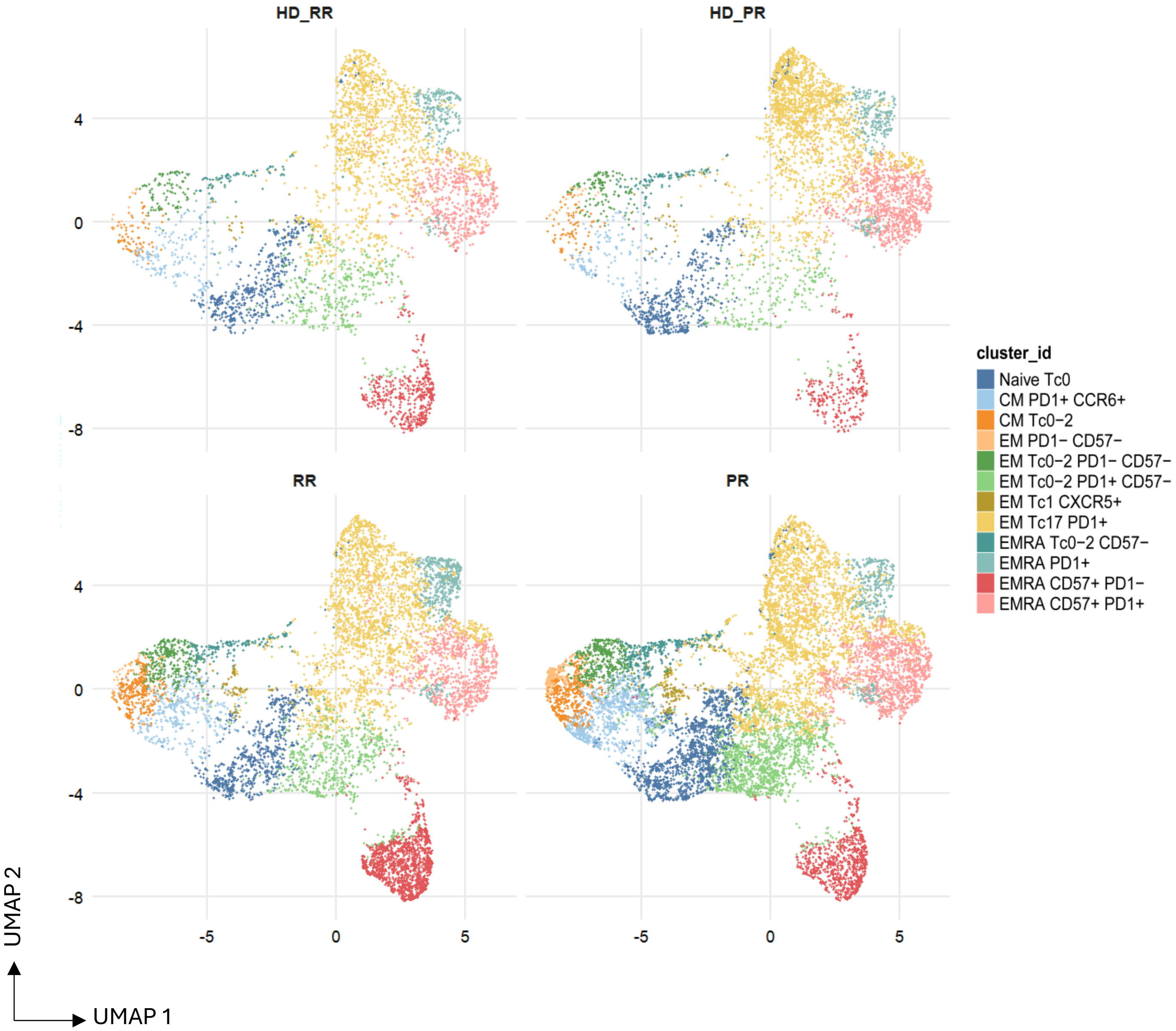


**Supplementary Figure S9.** Uniform Manifold Approximation and Projection (UMAP) plot shows the 2D spatial distribution of CD8 cells from 13 heallthy donors vaccinated against SARS-CoV2 and 23 patients with relapsing-remitting or progressive relapsing multiple sclerosis and vaccinated against COVID-19. UMAP graphs stratified by group**:** HD-PR: healthy donors-progressive relapsing (N=6); HD-RR: healthy donors-relapsing-remitting (N=7); PR: progressive relapsing (N=13); RR: relapsing-remitting(N=10).

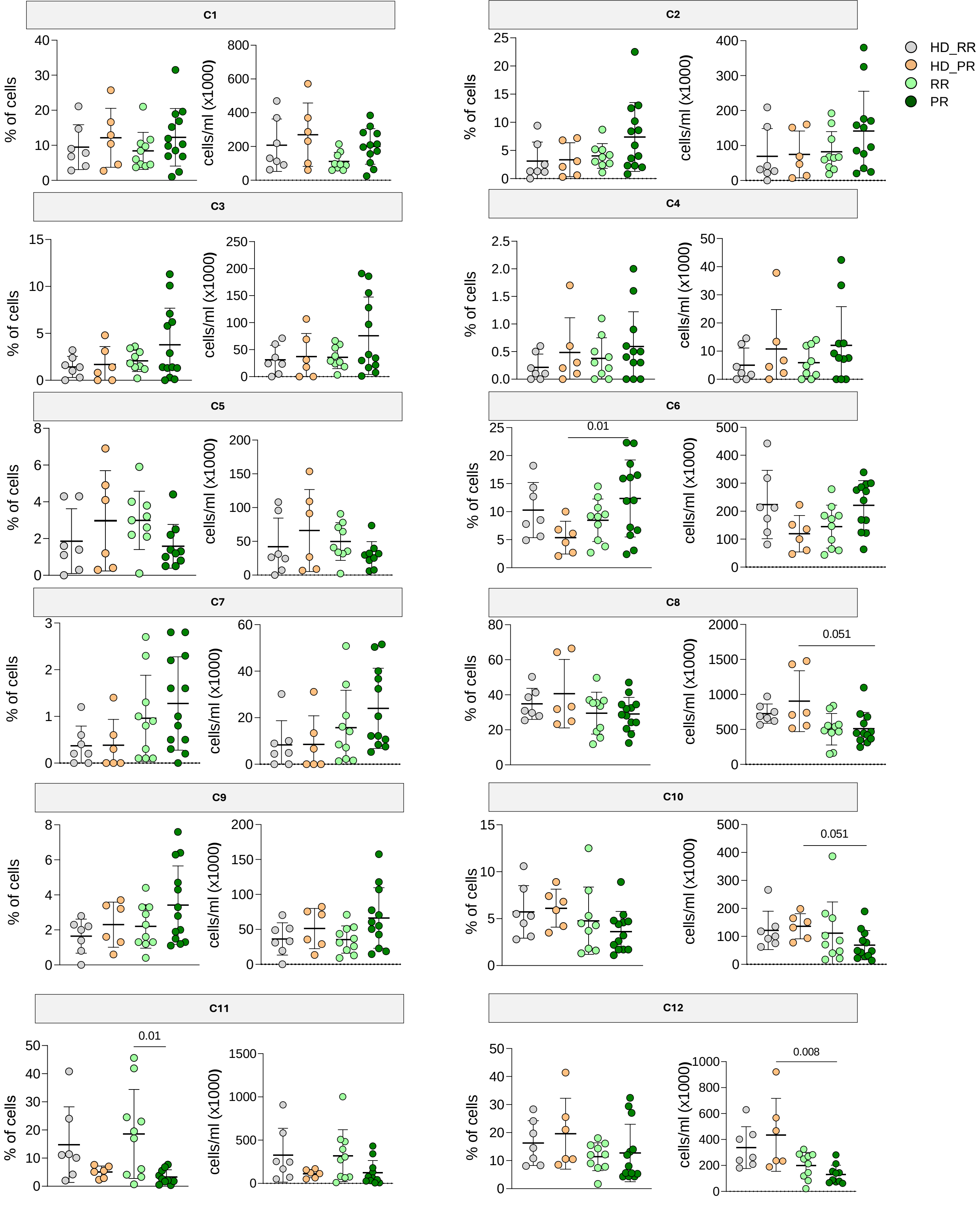


**Supplementary Figure S10.** Detailed statistical analysis of CD8⁺clusters obtained using FlowSOM. On the left, dot plots show the percentage of cells in different groups of MS patients (PR and RR) and healthy donors (HD-RR and HD-PR). On the right, dot plots show the absolute number of cells in different groups of patients and HD. Scatter plots show individual values; the central bar represents the mean ± SD. Kruskal–Wallis test (one-sided) with Benjamini–Hochberg correction for multiple comparisons. Tables display statistically significant q-value and individual p-value obtained. For all graphs: HD-PR: healthy donors-progressive relapsing (N=6); HD-RR: healy donors-relapsing-remitting (N=7); PR: progressive relapsing (N=13); RR: relapsing-remitting(N=10).

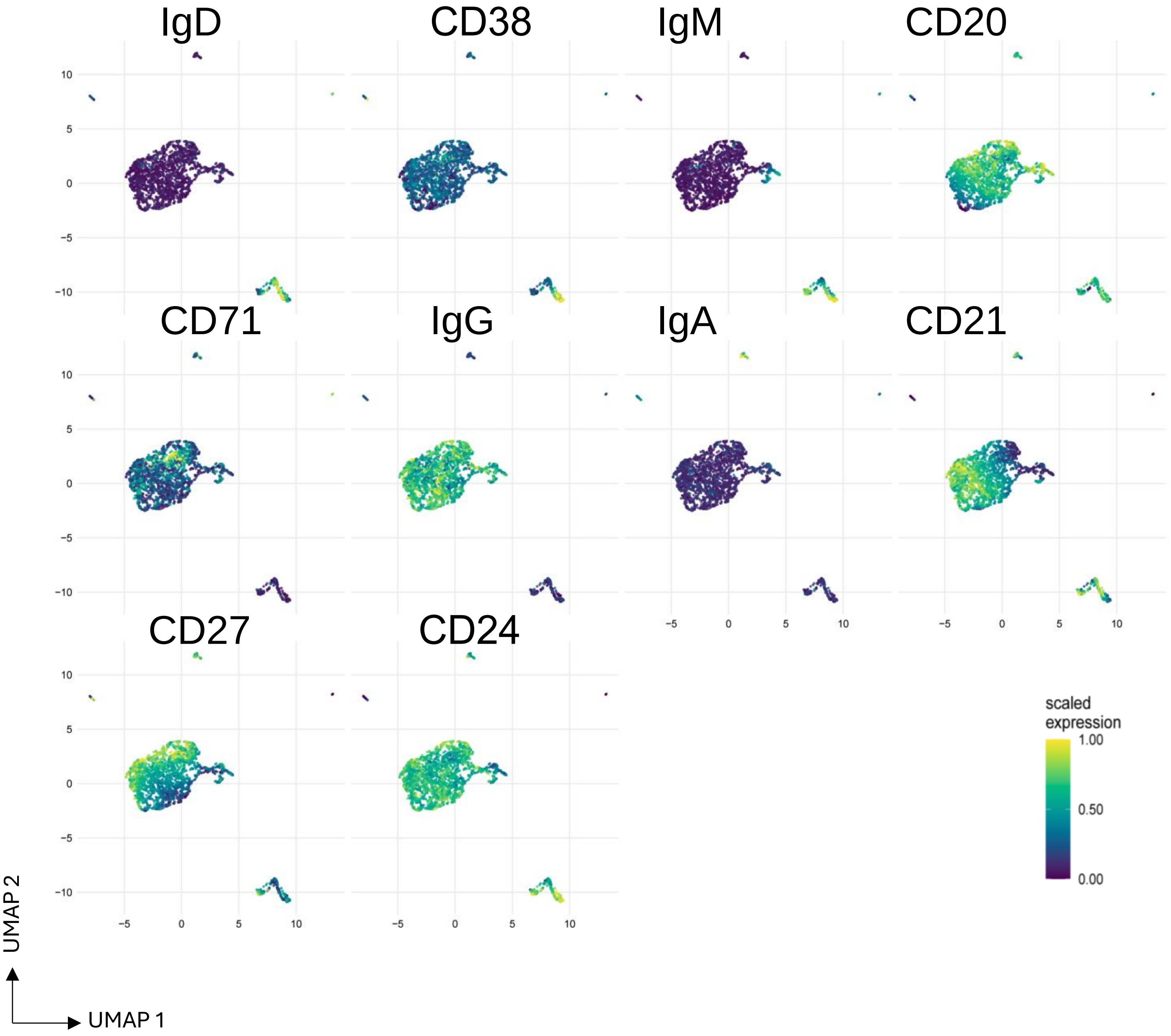


**Supplementary Figure S11.** Uniform Manifold Approximation and Projection (UMAP) plot shows the 2D spatial distribution of B cells from 13 heallthy donors vaccinated against SARS-CoV2 and 23 patients with relapsing-remitting or progressive relapsing multiple sclerosis and vaccinated against COVID-19. UMAP graphs colored by the expression of 10 markers used for $CD19^+$ antigen specific B cell phenotyping. Blue represents lower expression while yellow represent higher expression.

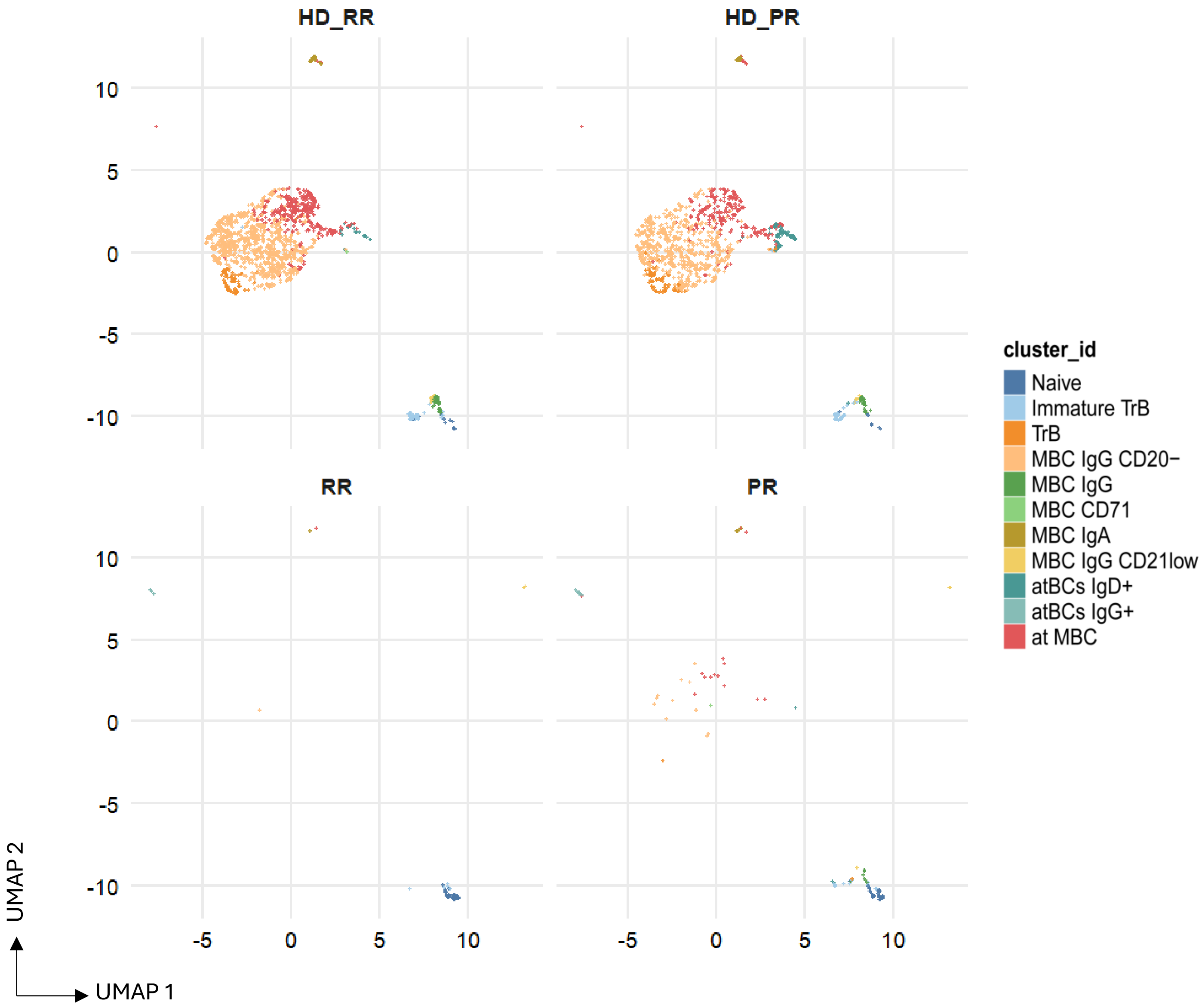


**Supplementary Figure S12.** Uniform Manifold Approximation and Projection (UMAP) plot shows the 2D spatial distribution of B cells from 13 heallthy donors vaccinated against SARS-CoV2 and 23 patients with relapsing-remitting or progressive relapsing multiple sclerosis and vaccinated against COVID-19. UMAP graphs stratified by group: HD-PR: healthy donors-progressive relapsing (N=6); HD-RR: healthy donors-relapsing-remitting (N=7); PR: progressive relapsing (N=13); RR: relapsing-remitting (N=10).
